# Epstein-Barr virus hijacks histone demethylase machinery to drive epithelial malignancy progression through KDM5B upregulation

**DOI:** 10.1038/s41392-025-02163-5

**Published:** 2025-03-10

**Authors:** Ya-Qing Zhou, Jia-Xin Jiang, Shuai He, Yi-Qi Li, Xi-Xi Cheng, Shu-Qiang Liu, Pan-Pan Wei, Xin-Yuan Guan, Choon Kiat Ong, Vivien Ya-Fan Wang, Chun-Ling Luo, Jin-Xin Bei

**Affiliations:** 1 State Key Laboratory of Oncology in South China, Guangdong Key Laboratory of Nasopharyngeal Carcinoma Diagnosis and Therapy, Guangdong Provincial Clinical Research Center for Cancer, Collaborative Innovation Center for Cancer Medicine, Sun Yat-sen University Cancer Center, Guangzhou, 510060 P.R. China; 2 Department of Clinical Laboratory, Sun Yat-sen University Cancer Center, Guangzhou, P.R. China; 3 Department of Experimental Research, Sun Yat-sen University Cancer Center, Guangzhou, P.R. China; 4 Department of Clinical Oncology, The University of Hong Kong, Hong Kong, P.R. China; 5 Lymphoma Translational Research Laboratory, Division of Cellular and Molecular Research, National Cancer Centre Singapore, Singapore, Singapore; 6 Cancer and Stem Cell Biology Program, Duke-NUS Medical School, Singapore, Singapore; 7 Faculty of Health, University of Macau, Avenida da Universidade, Taipa, Macau SAR, P.R. China; 8 Department of Medical Oncology, National Cancer Centre of Singapore, Singapore, Singapore; 9 Sun Yat-sen University Institute of Advanced Studies Hong Kong, Science Park, Hong Kong SAR, P.R. China; 10 Department of Clinical Oncology, School of Clinical Medicine, Li Ka Shing Faculty of Medicine, University of Hong Kong, Hong Kong SAR, P.R. China

**Keywords:** Cancer, Epigenetics

## Abstract

Epstein-Barr virus (EBV) is a significant epigenetic driver in the development of epithelial-origin nasopharyngeal carcinoma (NPC) and gastric cancer (GC), which together represent 80% of EBV-associated malignancies. Despite its known association, the specific mechanisms, particularly those involving EBV-induced histone modifications, remain poorly understood. Through integrative analyses of single-cell and bulk transcriptome data from epithelial tumor tissues and EBV-infected cells, we identified *KDM5B* as a critical histone-modifying factor consistently upregulated following EBV infection. We demonstrated that EBV stimulates *KDM5B* expression via interactions of its latent gene EBNA1 with transcription factor CEBPB and through direct binding of its lytic gene BZLF1 to Zta-response elements on the *KDM5B* promoter. Functional assays revealed that *KDM5B* acts as an oncogene, correlating with poor survival outcomes in EBV-associated epithelial cancers. Mechanistically, KDM5B inhibited the tumor suppressor gene *PLK2* through histone demethylation, thereby activating the PI3K/AKT/mTOR signaling pathway and promoting malignant progression. Furthermore, treatment with the KDM5B inhibitor AS-8351 markedly attenuated this signaling activity and exhibited strong anti-tumor effect in both in vitro and in vivo patient-derived xenograft models from EBV-associated tumors. Together, these findings provide novel insights into how EBV hijacks KDM5B to mediate histone demethylation of PLK2, facilitating tumor progression through the PI3K/AKT/mTOR pathway in epithelial cancers, highlighting promising therapeutic strategies targeting epigenetic alterations in EBV-associated cancers.

## Introduction

Epstein-Barr virus (EBV), a highly prevalent human herpesvirus infecting over 90% of the global population, is a critical etiological agent in the development of various malignancies.^1^ Among these, nasopharyngeal carcinoma (NPC) and EBV-associated gastric cancer (EBVaGC) are particularly significant due to their epithelial origin and substantial disease burden, collectively accounting for approximately 80% of all EBV-associated cancers.^2^ Although the association between EBV and these cancers is well-established by the presence of EBV molecules in tumors and elevated plasma EBV DNA levels during disease progression,^3^ the precise mechanisms through which EBV drivers cancer development remain incompletely understood.

EBV exhibits two distinct phases in its life cycle, namely latency and lysis,^4^ both of which are implicated in the oncogenesis of epithelial tumors.^5,6^ During latency, EBV expresses a specific set of genes, including EBV nuclear antigen 1 (*EBNA1*), latent membrane protein 1 (*LMP1*), and latent membrane protein 2 A (*LMP2A*). These viral proteins disrupt cellular processes, evade immune surveillance, and mediate malignant transformation.^5^ Although latent infection predominates in EBV-associated malignancies, lytic activation also plays a crucial role in tumorigenesis. Complete lytic replication, which is essential for viral transmission, enhances malignancy through mechanisms such as cytokine release, angiogenesis, and extracellular matrix modification.^7,8^ Furthermore, an incomplete lytic state characterized by the expression of early lytic genes without full virion production can induce genetic alterations, further facilitating tumor progression.^9,10^ Despite these findings, the specific mechanisms by which EBV’s latent and lytic cycles contribute to the tumorigenesis of epithelial malignancy remains to be fully elucidated.

Epigenetic regulation, particularly through histone modifications, plays a pivotal role in the initiation and progression of cancer.^11^ Histone modifications, such as methylation, acetylation, ubiquitination, and phosphorylation, alter chromatin structure and accessibility to activate or repress gene transcription.^12^ EBV infection in B cells leads to the loss of transcriptional repression-associated histone marks, including H3K9me3, H3K27me3, and H4K20me3, which enhances chromatin accessibility and regulates genes involved in cell cycle and apoptosis.^13^ In the context of EBV-associated malignancies, histone modifications are notably prevalent and can be driven by viral proteins, leading to the deregulation of host cellular pathways and contributing cancer development.^14^ EBV-encoded *LMP1* triggers *KDM6B* expression, leading to a reduction in H3K27me3 levels across the host genome in Hodgkin lymphoma.^15^ EBV infection reduces the transcriptionally active histone mark H3K4me3 and increases the repressive mark H3K27me3 at the promoter regions of genes involved in DNA damage repair process, resulting in their downregulation in immortalized nasopharyngeal epithelial cells.^16^ Additionally, EBV infection induces global alteration of histone modifications in EBVaGC cells, including the loss of repressive marks like H3K9me3 and H3K27me3 and gain of active marks such as H3K4me1 and H3K4me3, leading to aberrant enhancer activation.^17^ However, the specific histone-modifying factors involved in EBV-induced histone modification alterations and their carcinogenic mechanisms remain unclear in EBV-associated epithelial tumors.

In this study, we conduct integrative analyses of single-cell transcriptome data from NPC biopsy samples and bulk RNA-seq data from EBV-infected cell models, identifying lysine demethylase 5B (*KDM5B*) as a pivotal histone modification regulator involved in EBV pathogenesis. We further investigate the mechanisms by which EBV induces *KDM5B* expression and its impact on tumor progression, focusing on histone modifications and key signaling pathways. Our findings reveal how EBV exploits *KDM5B* to facilitate tumor progression through epigenetic modifications, specifically highlighting the suppression of tumor suppressors and the downstream signaling pathways. Our study provides novel insights into the epigenetic mechanisms underlying EBV-associated epithelial cancers and new therapeutic strategies targeting these epigenetic alterations.

## Results

### Identification of KDM5B as a crucial epigenetic factor in EBV-induced epithelial tumor progression

**Fig. 1:**
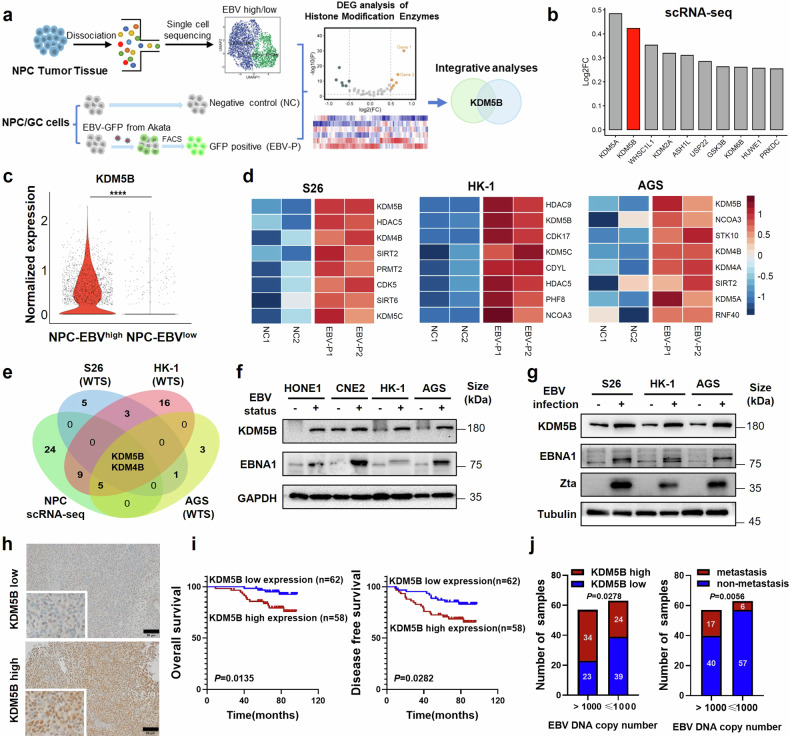
KDM5B is highly expressed and associated with poor survival in EBV-associated epithelial tumors. **a** Schematic diagram for the screening of histone modifier KDM5B contributing to EBV-associated epithelial tumors. FACS, Fluorescence Activated Cell Sorter. **b** Histogram showing top 10 upregulated histone modifiers in EBV^high^ NPC cell cluster, ranked according to their fold change in relative to the EBV^low^ cluster in scRNA-seq cohort (*n* = 10). **c** Violin plots depicting the normalized expression of KDM5B in EBV^high^ and EBV^low^ clusters from the NPC scRNA-seq data described in (**b**). **d** Heatmap illustrating top 8 upregulated histone modifiers in S26, HK-1, and AGS cells post-EBV infection. NC1/2, two replicate negative control groups; EBV-P1/2, two replicate EBV-GFP positive groups. Filled colors from blue to red represent scaled expression levels (normalized −log_10_*P* values) from low to high. *P*-values were calculated by one-sided hypergeometric test and adjusted for multiple comparisons. **e** Venn diagram showing the overlap between the datasets described in (**b**) and (**d**). Numbers are the upregulated genes. **f** Western blot assay quantifying the protein levels of KDM5B and EBNA1 in EBV-positive NPC (HONE1, CNE2, and HK-1) and GC (AGS) cell lines compared to their parental EBV-negative cells. GAPDH serves as a loading control. **g** Western blot assay demonstrating the protein levels of KDM5B, EBNA1, and Zta in NPC (S26 and HK-1) and GC (AGS) cell lines pre- and post-EBV infection. Tubulin is used as control. **h** Representative images of IHC staining for high (bottom) and low (top) protein levels of KDM5B in NPC samples (*n* = 120). **i** Survival analysis correlating KDM5B protein expression with overall (OS; left) and disease-free survival (DFS; right) in NPC patients detailed in (**h**). **j** Distribution of KDM5B expression levels (left) and metastasis rates (right) in NPC samples with EBV copy numbers greater than 1000 or not. KDM5B expression was determined using IHC staining scores. Comparison was done using Student’s t-test or one-way ANOVA with Bonferroni’s post-test. Data were presented as mean ± SD. *P < 0.05, **P < 0.01, ***P < 0.001, ****P < 0.0001. SD, standard deviation. Scale bar, 50 μm

To explore the histone modification mechanisms underlying EBV-induced epithelial tumor progression, we initially analyzed single-cell transcriptome data of EBV^high^ and EBV^low^ malignant epithelial cells from 10 NPC tissues (Fig. 1a).^18^ Differential gene expression analysis identified 40 histone modification enzymes that were upregulated in the EBV^high^ NPC cells as compared to the EBV^low^ cells (Fig. 1b, c and Supplementary Table 1), referencing with an online database of histone modifiers.^19^ Next, we established an in vitro EBV infection model using GFP-integrated EBV construct to infect two EBV-negative NPC cell lines (S26 and HK-1) and one EBV-negative GC cell line AGS (see Methods). EBV infected or positive (EBV-P) cells were sorted according to their GFP expression levels using flow cytometry, with un-infected cells serving as negative control (NC; Fig. 1a; see Methods). Subsequent transcriptome sequencing analysis of these cell groups pinpointed 11, 35, and 11 upregulated histone modification factors following EBV infection in S26, HK-1, and AGS cells, respectively (Fig. 1d and Supplementary Table 1). Integrative analysis of these histone modification factors identified *KDM5B* and *KDM4B* to be consistently upregulated in both biopsy samples and EBV infection models, with *KDM5B* exhibiting the most significant increase (Fig. 1e and Supplementary Table 1). Western blot analysis corroborated these findings, showing increased protein levels of KDM5B in EBV-positive NPC (HONE1, CNE2, and HK-1) and GC (AGS) cell lines compared to their parental EBV-negative cells (Fig. 1f). Notably, we observed a consistent rise in KDM5B protein expression in both the originally EBV-negative NPC (S26 and HK-1) and GC cell lines (AGS) following EBV infection (Fig. 1g). These findings strongly suggest the pivotal role of *KDM5B*, whose upregulation appears to be driven by EBV infection, in the progression of EBV-induced epithelial tumors.

We next evaluated the clinical relevance of *KDM5B*, utilizing immunohistochemistry (IHC) staining assays on specimens from 120 NPC patients (Fig. 1h). Our survival analysis demonstrated that elevated KDM5B expression was significantly correlated with both reduced overall survival (OS) and disease-free survival (DFS) in NPC patients (Fig. 1i). Moreover, upregulated KDM5B expression exhibited a strong association with metastasis in NPC patients (Supplementary Tables 2–3). Importantly, patients with high EBV DNA levels (>1000 copies/ml in plasma) demonstrated significantly higher KDM5B expression and an increased rate of metastasis compared to individuals with lower EBV levels (Fig. 1j). Taken together, these findings corroborate the importance of *KDM5B* in the progression of EBV-related epithelial tumors.

### EBNA1 cooperatively interacts with CEBPB to promote *KDM5B* transcription

**Fig. 2:**
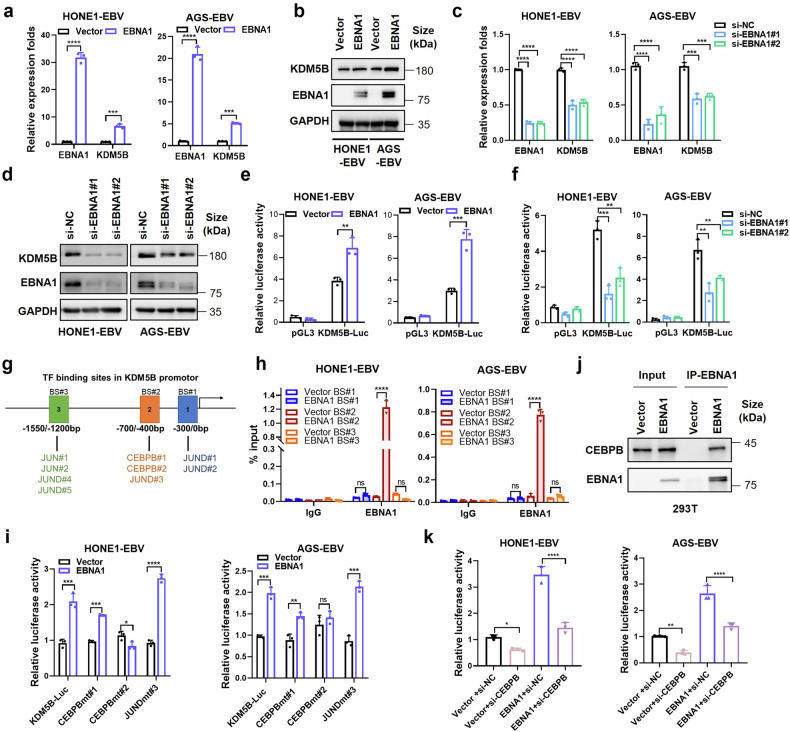
EBNA1 interacts with CEBPB to promote KDM5B expression. **a** RT-qPCR assay quantifying the expression of EBNA1 and KDM5B in EBV positive NPC (HONE1-EBV) and GC (AGS-EBV) cells infected with EBNA1-overexpressing or control vectors. **b** Western blot assay evaluating the protein levels of KDM5B and EBNA1 in cells described in (**a**), using GAPDH as a control. **c**, RT-qPCR analysis showing the expressions of EBNA1 and KDM5B expression in HONE1-EBV and AGS-EBV cells transfected with siRNA against EBNA1 (si-EBNA1#1 and si-EBNA1#2) or negative control (si-NC). **d** Western blot assay detecting the protein levels of KDM5B and EBNA1 in cells described in (**c**). GAPDH is used as control. **e**, **f** Luciferase assays measuring the transcriptional activity of KDM5B in HONE1-EBV and AGS-EBV cells transfected with EBNA1 or empty vector (**e**) and EBNA1 siRNAs or control siRNA (**f**), respectively. **g** Illustration of putative transcription factor binding sites (BS#1, BS#2, and BS#3) in the KDM5B promoter. **h** ChIP-qPCR analysis for BS#1, BS#2 and BS#3 at the KDM5B promotor following EBNA1 overexpression in HONE1-EBV and AGS-EBV cells. **i** Luciferase assays evaluating the KDM5B transcription activity in HONE1-EBV and AGS-EBV cells transfected with either EBNA1 overexpression constructs or empty vector, alongside KDM5B-Luc or mutated transcription factor binding site constructs (CEBPBmt#1, CEBPBmt#2, JUNDmt#3). **j** Western blot showing EBNA1 co-immunoprecipitation with CEBPB in 293 T cells. **k** Luciferase assays assessing the KDM5B transcription activity in HONE1-EBV and AGS-EBV cells transfected with the combinations of vector and siRNAs targeting CEBPB or control (si-NC), in the presence or absence of EBNA1. Statistical analysis was conducted using Student’s *t*-test for two groups and one-way ANOVA followed by Dunnett’s post hoc test for more than two groups, with data presented as mean ± SD. *P < 0.05, **P < 0.01, ***P < 0.001, ****P < 0.0001. ns, no significance. SD, standard deviation

Considering the typical latency of EBV within EBV-related epithelial tumors,^5^ we investigated how EBV infection influences *KDM5B* expression in these malignancies. We first examined the correlation of expression between *KDM5B* and all EBV genes, utilizing transcriptome data from 113 NPC tumor biopsy samples. Notably, we observed a unique positive correlation between *KDM5B* and *EBNA1* in NPC tumor samples (Supplementary Fig. 1a), collaborating with the concurrent upregulation of KDM5B and EBNA1 in EBV-positive or EBV-infected NPC and GC cells (Fig. 1f, g). Next, we separately introduced the EBNA1, together with other two classical EBV latent genes LMP1 and LMP2A, into EBV-positive NPC (HONE1-EBV, C666-1) and GC (AGS-EBV) cells. Real-time quantitative PCR (RT-qPCR) analysis demonstrated that *EBNA1* overexpression significantly increased *KDM5B* expression at both mRNA and protein levels (Fig. 2a, b and Supplementary Fig. 1b), a pattern not observed in cells with *LMP1* and *LMP2A* overexpression (Supplementary Fig. 1c, d). By contrast, *EBNA1* knockdown led to a notable decrease in *KDM5B* expression (Fig. 2c, d and Supplementary Fig. 1e). These observations strongly suggest a specific regulatory relationship between *EBNA1* and *KDM5B*. Considering EBNA1’s role as a transcriptional activator in EBV-associated tumors,^20^ we conducted luciferase reporter assay, revealing that *EBNA1* overexpression significantly enhanced *KDM5B* transcription activity in NPC and GC cells, while its knockdown resulted in reduced transcription activity (Fig. 2e, f and Supplementary Fig. 1f, g). These findings underscore EBNA1’s regulatory effect on *KDM5B* transcription, highlighting a specific epigenetic mechanism within EBV-induced epithelial tumor progression.

To uncover the regulatory mechanisms of EBNA1 on *KDM5B* transcription, we examined the presence of EBNA1 binding motif within *KDM5B* promoter region, which spans −2 kb to +2 kb distance to the transcription start site (TSS). However, no identifiable EBNA1 binding motif was observed, suggesting an indirect regulation role of EBNA1 on *KDM5B* transcription. Given EBNA1’s known capacity to interact with various cofactors for DNA binding and gene transcription regulation,^21^ we hypothesized that EBNA1 could facilitate *KDM5B* transcription through interactions with other transcription factors (TFs). By leveraging the hTFtarget database, we identified 188 TFs with potential binding affinity to the *KDM5B* promoter. Subsequent correlation analysis using the scRNA-seq data from 10 NPC samples and bulk-seq data from 113 NPC samples identified 14 TFs positively correlated with *KDM5B* expression, with *CEBPB*, *JUND*, and *JUN* showing particularly strong correlations (Fig. 2g and Supplementary Fig. 1h). Chromatin immunoprecipitation combined with quantitative PCR (ChIP-qPCR) analysis demonstrated that EBNA1 binds to a specific region (−700 bp/−400 bp upstream of the TSS) in the *KDM5B* promoter in both HONE1-EBV and AGS-EBV cells (Fig. 2h), which encompasses binding sites (BS) for CEBPB#1, CEBPB#2 and JUND#3 (Fig. 2g, h). Luciferase reporter assays indicated that mutations at the CEBPB#2 binding site specifically abrogated EBNA1’s ability to activate *KDM5B* transcription (Fig. 2i), suggesting a pivotal role of *CEBPB* in this regulatory mechanism. This was further supported by co-immunoprecipitation assays, confirming the interaction between EBNA1 and CEBPB (Fig. 2j and Supplementary Fig. 1i). Moreover, *CEBPB* knockdown not only decreased *KDM5B* transcription but also diminished the EBNA1-induced *KDM5B* upregulation (Fig. 2k). Additionally, overexpression of EBNA1 mutant without the functional DNA-binding domain (DBD) still increased KDM5B expression, with effect comparable to the intact EBNA1 (Supplementary Fig. 1j), which further supports the indirect regulation of EBNA1 on *KDM5B* transcription. Collectively, these findings elucidate a mechanism by which EBNA1 activates *KDM5B* transcription through a cooperative interaction with CEBPB.

To further investigate whether EBNA1 regulation of *KDM5B* is dependent on the presence of EBV, we first performed ChIP-qPCR experiments with EBNA1 antibody in EBNA1-negative wild-type HONE1 and AGS cells, revealing no significant enrichment of EBNA1 on the CEBPB#1, CEBPB#2 and JUND#3 binding sites in KDM5B promoter (Supplementary Fig. 1k). Moreover, EBNA1 overexpression did not affect *KDM5B* expression in EBV-negative cells (HK-1, AGS and 293 T; Supplementary Fig. 1l, m). Taken together, these observations suggest that EBNA1-mediated regulation of *KDM5B* is EBV-dependent and likely involves other EBV-encoded factors.

### BZLF1 directly activates *KDM5B* transcription via binding to its ZREs

**Fig. 3:**
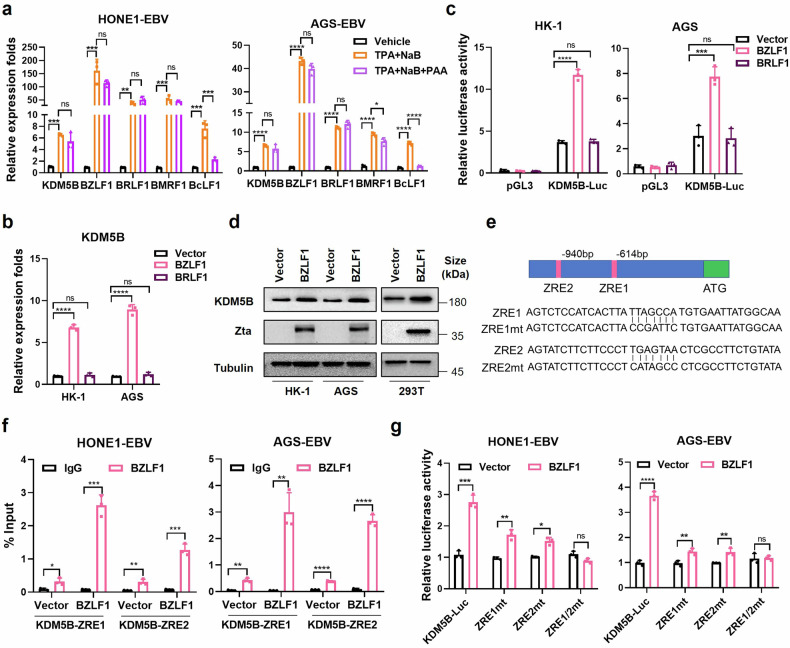
BZLF1 activates KDM5B expression via direct binding with the ZREs on its promoter. **a** RT-qPCR analysis evaluating the mRNA expressions of BZLF1, BRLF1, BMRF1, BcLF1, and KDM5B in HONE1-EBV and AGS-EBV cells treated with TPA + NaB, TPA + NAB + PAA, or vehicle control. **b** RT-qPCR analysis showing KDM5B expression in EBV negative NPC (HK-1) and GC (AGS) cells transfected with BZLF1-, BRLF1-overexpressing or empty vectors. **c** Luciferase assays quantifying the KDM5B transcription activity in HK-1 and AGS cells transfected with BZLF1-, BRLF1-overexpressing or empty vectors. **d** Western blot assay measuring the protein level of KDM5B in HK-1, AGS, and 293 T cells transfected with BZLF1-overexpressing or empty vectors. **e** Schematic illustration of BZLF1 response elements (ZRE1 and ZRE2) at KDM5B promotor (upper) and their corresponding mutants (ZRE1mt, ZRE2mt, ZRE1/2 mt, bottom) employed in ChIP-qPCR and luciferase assay in (**f**) and (**g**). **f** ChIP-qPCR analyzing the binding of BZLF1 on ZRE1 and ZRE2 at KDM5B promotor, before and after BZLF1 overexpression in HONE1-EBV and AGS-EBV cells. **g** Luciferase assays evaluating the KDM5B activity in HONE1-EBV and AGS-EBV cells transfected with either KDM5B-Luc or mutant reporter plasmids corresponding to ZRE binding sites (ZRE1mt, ZRE2mt, ZRE1/2mt), following vector or BZLF1 overexpression. Statistical analysis is performed using Student’s *t*-test for two groups and one-way ANOVA followed by Dunnett’s post hoc test or Sidak’s post hoc test for more than two groups. Results are presented as the mean ± SD. *P < 0.05, **P < 0.01, ***P < 0.001, ****P < 0.0001. ns, no significance. SD, standard deviation

Considering the role of EBV lytic cycle in tumor progression,^22^ we next investigated the potential activation of *KDM5B* during EBV lytic replication, through treating EBV-positive cell lines with phorbol 12-myristate 13-acetate (TPA) and sodium butyrate (NaB), reagents known to induce EBV lytic reactivation.^23^ RT-qPCR confirmed an increase in lytic gene expression (*BZLF1*, *BRLF1*, *BMRF1*, and *BcLF1*) in EBV-positive NPC and GC cells treated with TPA and NaB compared to control group (Fig. 3a and Supplementary Fig. 2a). Notably, a significant upregulation of *KDM5B* was detected in these cells (Fig. 3a and Supplementary Fig. 2a, b), indicating that EBV lytic activation promotes *KDM5B* transcription.

EBV lytic cycle includes the expression of early lytic genes (immediate early and early), DNA replication, expression of late lytic genes, and finally, virus assembly and release.^4^ To pinpoint the specific phase of lytic replication responsible for the surge in *KDM5B* expression, we introduced phosphonoacetic acid (PAA), an inhibitor of EBV DNA replication, into the treatment regimen. As expected, PAA treatment led to a notable decrease in the expression of the late lytic gene *BcLF1* (Fig. 3a and Supplementary Fig. 2a), highlighting the essential role of the DNA replication phase in the transcription of late lytic genes.^4^ Intriguingly, despite PAA treatment, the elevated expression levels of *KDM5B* and early/intermediate lytic genes initially induced by TPA and NaB remained unaffected (Fig. 3a and Supplementary Fig. 2a). This suggests that *KDM5B* upregulation is specifically associated with the activation of early lytic genes during EBV reactivation, independent of the DNA replication phase.

*BZLF1* (encoding Zta) and *BRLF1* (encoding Rta) are immediate early lytic genes pivotal for initiating the lytic gene expression cascade by stimulating lytic EBV promoters.^4^ Given the undetectable expression of these two lytic genes in most NPC samples, no correlation was observed between *BZLF1* or *BRLF1* and *KDM5B* (Supplementary Fig. 1a). We next overexpressed *BZLF1* and *BRLF1* in NPC and GC cells to assess the impact of these genes on *KDM5B* activation. RT-qPCR and luciferase reporter assays demonstrated that BZLF1, but not BRLF1, significantly enhanced *KDM5B* expression in EBV-negative cells (Fig. 3b, c). This effect of BZLF1 was consistently observed in EBV-positive cells, where BZLF1 overexpression led to a more than 10–15 folds increase in KDM5B expression, while BRLF1 overexpression resulted in only a slight increase compared to control cells (Supplementary Fig. 2c, d). Additionally, western blot assay confirmed that BZLF1 overexpression increased the protein level of KDM5B in EBV-negative HK-1 and AGS cells, as well as 293 T cells (Fig. 3d). These observations collectively underscore BZLF1 as a crucial modulator of *KDM5B* expression during EBV lytic replication.

To probe how BZLF1, or Zta, regulates *KDM5B* expression, we examined potential Zta-response elements (ZREs), where BZLF1 binds to regulate gene expression,^22^ within the *KDM5B* promoter. Intriguingly, we identified two ZREs located at −614 ~ −621 bp (ZRE1) and −940 ~ −947 bp (ZRE2) regions upstream of the TSS in *KDM5B* (Fig. 3e). To verify the direct interaction between BZLF1 and the *KDM5B* promoter, we conducted ChIP-qPCR assay with BZLF1-specific antibodies in HONE1-EBV and AGS-EBV cell lines. This assay revealed significant binding of BZLF1 to both the ZRE1 and ZRE2 regions (Fig. 3f), indicating a direct regulatory relationship. Moreover, luciferase assays revealed that individual mutations in ZRE1 (ZRE1mt) or ZRE2 (ZRE2mt) partially reduced the BZLF1-induced activation of *KDM5B* transcription, whereas simultaneous mutations in both ZREs regions (ZRE1/2mt) completely abrogated this effect (Fig. 3g and Supplementary Fig. 2e). Together with the KDM5B upregulation by BZLF1 observed in EBV-negative cells (Fig. 3b, c), these findings establish that BZLF1, an immediate early EBV lytic gene, directly stimulates *KDM5B* expression by binding to the ZREs within the *KDM5B* promoter, independent of EBV infection status.

Given the proximity of the EBNA1 and BZLF1 binding sites on the *KDM5B* promoter (only 55 bp apart), we wondered whether the regulatory effects of EBNA1 and BZLF1 on *KDM5B* are independent. To test this, we conducted luciferase assays to evaluate the impact of BZLF1 and EBNA1 on the full-length *KDM5B* promoter, as well as various mutant constructs, including CEBPBmt#1, CEBPBmt#2, JUND#3, ZRE1mt, ZRE2mt, and ZRE1/2mt, in HONE1-EBV and AGS-EBV cell lines. Our results showed that EBNA1 overexpression enhanced the transcriptional activity of the *KDM5B* promoter even in the presence of ZRE mutations (Supplementary Fig. 2f). Similarly, BZLF1 overexpression increased the transcriptional activity of the *KDM5B* promoter containing mutations in the CEBPB and JUND binding motifs (Supplementary Fig. 2g). These findings strongly suggest that the transcriptional activation of *KDM5B* by EBNA1 and BZLF1 occurs independently.

### *KDM5B* plays an oncogenic role in EBV-associated epithelial tumors

**Fig. 4:**
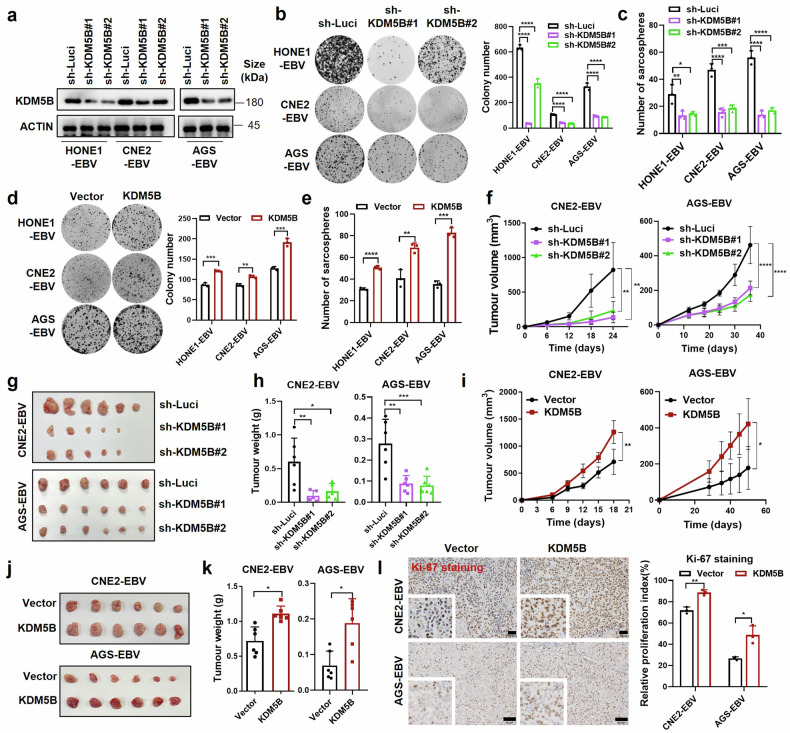
Oncogenic role of KDM5B in EBV-associated epithelia tumor cells. **a** Western blot assay confirming the knockdown efficiency of KDM5B in HONE1-EBV, CNE2-EBV, and AGS-EBV cells infected with lentivirus expressing KDM5B shRNAs (sh-KDM5B#1 and sh-KDM5B#2) or control shRNA (sh-Luci). ACTIN serves as a loading control. **b** Colony formation ability of cells described in (**a**), with statistics shown at the right. **c** Sphere formation ability of cells described in (**a**). **d** Colony formation results for HONE1-EBV, CNE2-EBV, and AGS-EBV cells infected with lentivirus expressing KDM5B or empty vector, along with corresponding statistics shown at the right. **e** Sphere formation ability of cells described in (**d**). **f** Growth curve of xenograft tumors derived from CNE2-EBV and AGS-EBV cells stably expressing KDM5B shRNAs (sh-KDM5B#1 and sh-KDM5B#2) or control shRNA (sh-Luci). Tumor size (**g**) and weight (**h**) for the xenograft tumors excised from (**f**). **i** Growth curve of xenograft tumors from CNE2-EBV and AGS-EBV cells stably expressing KDM5B or empty vector. Tumor size (**j**) and weight (**k**) for the xenograft tumors excised from (**i**). **l** Representative images for IHC staining of Ki-67 in xenograft tumors from CNE2-EBV and AGS-EBV cells with either KDM5B overexpression or control, with statistics summarized at the right. Statistical analysis is conducted using Student’s *t*-test for two groups and one-way ANOVA followed by Dunnett’s post hoc test for more than two groups, with data are presented as the mean ± SD. *P < 0.05, **P < 0.01, ***P < 0.001, ****P < 0.0001. SD, standard deviation. Scale bar, 50 μm

To investigate the role of *KDM5B* in EBV-associated epithelial tumors, we performed shRNA-mediated knockdown of *KDM5B* in EBV-positive NPC and GC cells (Fig. 4a). Cell growth and colony formation assays illustrated a significant decline in cell proliferation upon *KDM5B* knockdown (Fig. 4b and Supplementary Fig. 3a). Furthermore, sphere formation assays demonstrated a significant reduction in tumorigenic capacity of *KDM5B*-knockdown cells (Fig. 4c and Supplementary Fig. 3b), accompanied by their decreased expression of key stemness markers^24^ (*SOX2*, *NANOG*, *OCT4*, *ABCG2*, and *BMI-1*; Supplementary Fig. 3c). In contrast, *KDM5B* overexpression in tumor cells led to enhanced cell proliferation, colony formation, and sphere formation capacities (Fig. 4d, e and Supplementary Fig. 3d–f), alongside with increased levels of stemness markers (Supplementary Fig. 3g). These findings collectively underscore the essential role of *KDM5B* in promoting the oncogenic features of EBV-related epithelial tumors.

We next explored the tumorigenic function of *KDM5B* with in vivo mouse model, utilizing xenografts implanted with EBV-positive epithelial tumor cells that had been genetically modified to either stably knock down or overexpress *KDM5B*. Notably, xenografts derived from *KDM5B*-knockdown cells exhibited significantly reduced growth, as evidenced by decreased tumor volume and weight (Fig. 4f–h), and corroborated by diminished Ki-67 staining, indicative of low cell proliferation (Supplementary Fig. 3h). Conversely, xenografts from cells overexpressing *KDM5B* showed enhanced tumorigenic capacity and a higher proportion of Ki-67 positive cells (Fig. 4i–l). This in vivo evidence further emphasizes the pivotal oncogenic function of *KDM5B* in the progression of EBV-positive epithelial tumors.

### KDM5B suppresses *PLK2* expression through H3K4me3 demethylation

**Fig. 5:**
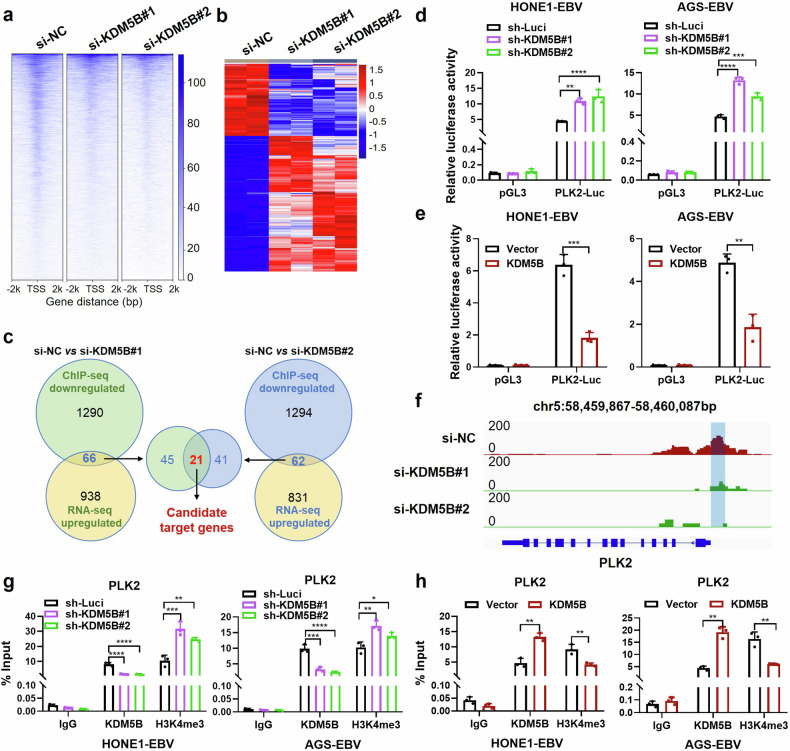
KDM5B represses PLK2 expression through H3K4me3 demethylation at its promoter. **a** ChIP-seq enrichment analysis showing KDM5B occupancy around the transcription start site (TSS) of target genes, ranging from -2 kilobases (kb) to +2 kb, following KDM5B knockdown (si-KDM5B#1/2), alongside with control (si-NC). **b** Heatmap showing the differential expressed genes (DEG) affected by KDM5B knockdown (si-KDM5B#1/2). Filled colors from blue to red represent expression fold change from low to high, in the KDM5B knockdown group compared to the control group. **c** Scheme diagram illustrating the integrative analysis of ChIP-seq and RNA-seq data for two KDM5B knockdown sets, followed by intersecting their shared genes. Luciferase assays evaluating the PLK2 transcription activity in HONE1-EBV and AGS-EBV cells with KDM5B knockdown (**d**) by shRNAs (#1/#2) or overexpression (**e**). **f** ChIP-Seq profiling KDM5B occupying signals in PLK2 loci following KDM5B knockdown by siRNAs (#1/2). ChIP-qPCR analysis evaluating the binding of KDM5B and H3K4me3 at PLK2 promotor in HONE1-EBV and AGS-EBV cells with KDM5B knockdown by shRNAs (**g**) or overexpression (**h**). Statistical analysis is conducted using Student’s *t*-test for two groups and one-way ANOVA followed by Dunnett’s post hoc test for more than two groups. Data are presented as the mean ± SD. *P < 0.05, **P < 0.01, ***P < 0.001, ****P < 0.0001. SD, standard deviation

To investigate the molecular mechanisms by which KDM5B contributes to tumor progression, we conducted a ChIP-seq analysis to map KDM5B occupancy in HONE1-EBV cells with targeted *KDM5B* knockdown (using siRNA targeting *KDM5B*, referred to as si-KDM5B#1/2; see Methods). This analysis revealed a significant presence of KDM5B across promoter regions at genome-wide level (Supplementary Fig. 4a), with a notable reduction in KDM5B occupancy around the TSS (−2 kb to +2 kb) following *KDM5B* knockdown (Fig. 5a). Transcriptome analysis identified 1943 and 1708 differentially expressed genes in two groups of NPC cells with *KDM5B* knockdown (Fig. 5b). Due to KDM5B’s function as a transcriptional repressor,^25^ our investigation focused on genes that were upregulated and contained decreased KDM5B occupancy in their promoters following *KDM5B* knockdown. This approach led to the identification of 21 genes potentially regulated by KDM5B (Fig. 5c). Further transcriptome analysis revealed eight genes (*FAT4*, *FHL2*, *GCLM*, *JPH2*, *IL17RE*, *SH3RF2*, *PLK2*, and *TPM4*) to be consistently downregulated in EBV-infected S26 and HK-1 NPC cells compared to uninfected cells (Data not shown). RT-qPCR analyses in both EBV-negative and -positive epithelial tumor cells, as well as those cells with *KDM5B* knockdown or overexpression, pinpointed *PLK2* as the primary target with the most significant alterations affected by *KDM5B* (Supplementary Fig. 4b–d). Luciferase reporter assays further corroborated these results, demonstrating that *KDM5B* knockdown markedly enhanced, whereas its overexpression reduced, *PLK2* transcription activity in HONE1-EBV and AGS-EBV cells (Fig. 5d, e).

Given that KDM5B exerts its transcriptional repression through removal of the trimethyl modification of H3K4 (H3K4me3, a marker for transcriptional activation),^25^ we assessed its role in activating *PLK2* promoter. ChIP-seq analysis demonstrated a substantial reduction in KDM5B occupancy in the −500 bp to −200 bp region upstream of the *PLK2* TSS in NPC cells upon *KDM5B* knockdown (Fig. 5f). Furthermore, ChIP-qPCR with KDM5B and H3K4me3 antibodies revealed that *KDM5B* knockdown reduced its occupancy and enhanced H3K4me3 modification at *PLK2* promoter in both NPC and GC cells (Fig. 5g). In contrast, *KDM5B* overexpression led to increased KDM5B occupancy and a decreased H3K4me3 modification at the promoter (Fig. 5h). These findings collectively indicate that KDM5B downregulates *PLK2* expression through reducing H3K4 trimethylation at the *PLK2* promoter. Additionally, we also observed a significant reduction in KDM5B enrichment and a corresponding increase in H3K4me3 enrichment at the promoter regions of several other candidate genes, including *FAT4*, *FHL2*, *GCLM*, *JPH2*, *IL17RE*, *SH3RF2*, and *TPM4*, following KDM5B knockdown (Supplementary Fig. 4e). These results are consistent with the ChIP-seq analysis (Supplementary Fig. 5), highlighting the critical role of KDM5B in regulating global histone modification processes.

### KDM5B facilitates tumor progression through suppressing *PLK2* in EBV-associated epithelial tumors

**Fig. 6:**
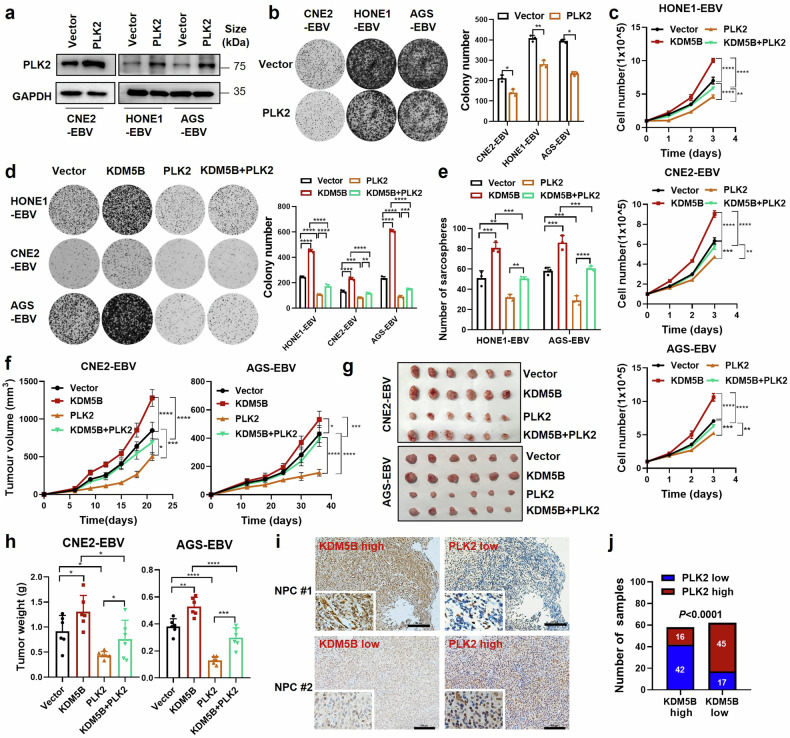
PLK2 mediates KDM5B-promoted tumor progression in EBV-associated epithelial tumors. **a** Western blot analysis showing the protein level of PLK2 in CNE2-EBV, HONE1-EBV, and AGS-EBV cells infected with lentivirus expressing PLK2 or empty vector. GAPDH is used as control. **b** Colony formation ability of cells described in (**a**), with statistics summarized at the right. **c** Cell growth curves of HONE1-EBV, CNE2-EBV, and AGS-EBV cells stably overexpressing KDM5B (KDM5B) or not (control vector), followed by infection of lentivirus expressing PLK2 or empty vector. **d** Colony formation ability of cells described in (**c**), with statistics presented at the right. **e** Sphere formation ability of HONE1-EBV and AGS-EBV cells described in (**c**). **f** Tumor growth curve of xenograft from CNE2-EBV and AGS-EBV cells described in (**c**). Tumor size (**g**) and weight (**h**) for the xenograft tumors excised from (**f**). **i** Representative IHC staining images showing the protein levels of KDM5B and PLK2 in NPC samples (NPC #1 and NPC #2). **j** Comparison of PLK2 expression level in NPC patients (*n* = 120) with high or low KDM5B expression based on IHC staining scores. Statistical analysis is conducted by Student’s *t*-test for two groups and one-way ANOVA followed by Sidak’s post hoc test for more than two groups. Data are presented as the mean ± SD. *P < 0.05, **P < 0.01, ***P < 0.001, ****P < 0.0001. SD, standard deviation. Scale bar, 100 μm

To explore the role of *PLK2* in EBV-related cancers, we first examined its effects on NPC and GC cell lines. We observed that *PLK2* overexpression significantly inhibited, while its knockdown notably enhanced, both growth and colony formation in EBV-positive epithelial tumor cells (Fig. 6a, b and Supplementary Fig. 6a–d). This aligns with the well-known tumor suppressor function of *PLK2* in various human cancers.^26,27^ To further investigate whether PLK2 counteracts KDM5B-driven tumor progression, we introduced simultaneous overexpression or knockdown of KDM5B and PLK2 in HONE1-EBV, CNE2-EBV and AGS-EBV cells. Intriguingly, *PLK2* overexpression remarkably attenuated the increased cell growth, colony formation, and sphere formation capacities of *KDM5B*-overexpressing EBV-positive NPC and GC cells as compared to the control groups (Fig. 6c–e and Supplementary Fig. 7a). Furthermore, KDM5B knockdown significantly inhibited NPC cell proliferation, while the suppressive effects were largely rescued by PLK2 knockdown (Supplementary Fig. 7b–d), suggesting that PLK2 is a major downstream mediator of KDM5B-induced tumor progression.

Moreover, in vivo xenograft models clearly showed that *PLK2* overexpression significantly curtailed the tumor growth boosted in CNE2-EBV and AGS-EBV cells due to *KDM5B* overexpression (Fig. 6f–h and Supplementary Fig. 7e). These observations highlight the capacity of PLK2 to counterbalance the tumorigenic function of KDM5B. Further IHC staining analyses demonstrated a notable negative correlation between PLK2 and KDM5B expression levels in an NPC cohort (*n* = 120; Fig. 6i, j). Kaplan-Meier survival analysis revealed a significant association between elevated PLK2 expression and improved overall and disease-free survival (Supplementary Fig. 7f), contrasting with the adverse association of KDM5B overexpression. Notably, patients with concurrent high KDM5B and low PLK2 expression exhibited the worst outcomes in terms of both OS (left) and DFS (right) survivals (Supplementary Fig. 7g). These findings strongly suggest that KDM5B may facilitate tumor progression of EBV-associated epithelial tumors by suppressing PLK2.

To investigate if the effects of KDM5B and PLK2 are EBV-dependent, we conducted parallel knockdown and overexpression experiments in EBV-negative HONE1, CNE2, and AGS cell lines. Our results demonstrated that KDM5B knockdown had a significantly weaker suppressive effect on cell proliferation in EBV-negative cells compared to EBV-positive cells (Supplementary Fig. 8a, b). Similarly, PLK2 knockdown induced a smaller proliferative effect in EBV-negative cells (Supplementary Fig. 8a, b). Notably, western blot analysis revealed that KDM5B knockdown in EBV-negative cells did not result in PLK2 upregulation (Supplementary Fig. 8c), a phenomenon observed in EBV-positive cells (Supplementary Fig. 7b). These findings strongly suggest that the regulation of PLK2 by KDM5B is dependent on the presence of EBV. Moreover, weaker effects of both KDM5B and PLK2 overexpression on cell proliferation were observed in EBV-negative cells compared to EBV-positive cells (Supplementary Fig. 8d–f). Collectively, these results underscore the critical role of the KDM5B/PLK2 axis in EBV-driven tumor progression and highlight its potential as therapeutic target in EBV-associated epithelial tumors.

### KDM5B/PLK2 axis mediate EBV-induced activation of the PI3K/AKT/mTOR pathway

**Fig. 7:**
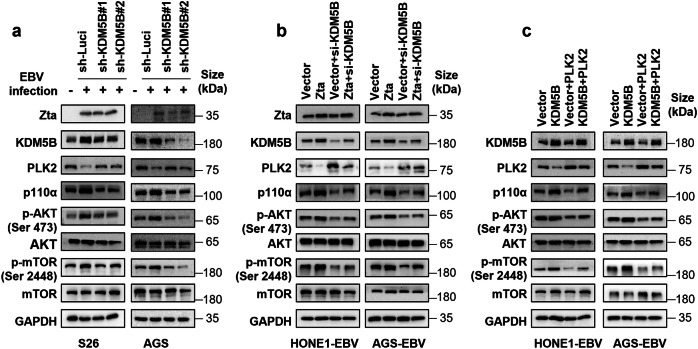
EBV-driven KDM5B activates the PI3K/AKT/mTOR pathway through PLK2 inhibition. **a** Western blot analysis determining the protein levels of Zta, KDM5B, PLK2, p110α, p-AKT (Ser 473), AKT, p-mTOR (Ser 2448), and mTOR in parental or EBV-infected S26 and AGS cells together with KDM5B knockdown by shRNAs or not. GAPDH served as the loading control. **b** Western blot assay showing the protein expressions of the indicated proteins in HONE1-EBV and AGS-EBV cells, transfected with either Zta or empty vector, followed by subsequent transfection of siRNA targeting KDM5B. GAPDH is used as a loading control. **c** Western blot assay showing the protein expressions of the indicated proteins in HONE1-EBV and AGS-EBV cells with stable overexpression KDM5B or an empty vector, followed by infection with lentivirus overexpressing PLK2. GAPDH is employed as a control

Given the pivotal role of the PI3K/AKT/mTOR signaling pathway in the progression of EBV-associated epithelial cancers,^28,29^ we investigated the effects of KDM5B and PLK2 on this pathway in NPC and GC cells. Western blot analyses revealed that EBV infection led to KDM5B upregulation and concurrently PLK2 downregulation in S26 and AGS cells, alongside with the PI3K/AKT/mTOR pathway activation reflected by the increased levels of p110α (a catalytic subunit of PI3K), p-AKT (Ser 473), and p-mTOR (Ser 2448) (Fig. 7a and Supplementary Fig. 9a). In contrast, *KDM5B* knockdown resulted in PLK2 upregulation and decreased the expression of these key signaling molecules in these cells upon EBV infection (Fig. 7a and Supplementary Fig. 9a). Moreover, while Zta initiated the activation of PI3K/AKT/mTOR pathway in HONE1-EBV and AGS-EBV cells, *KDM5B* knockdown abolished the Zta-induced effects on this signaling cascade (Fig. 7b and Supplementary Fig. 9b). Conversely, *KDM5B* overexpression led to the activation of the signaling cascade in these cells (Fig. 7c and Supplementary Fig. 9c), consistent with the finding in prostate tumor progression.^30^ However, introducing *PLK2* overexpression alongside *KDM5B* overexpression attenuated this activation (Fig. 7c and Supplementary Fig. 9c). These observations collectively suggest that EBV-induced KDM5B upregulation promotes PI3K/AKT/mTOR signaling through suppressing PLK2 in EBV-associated epithelial tumors.

### KDM5B inhibition attenuates tumor progression of EBV-positive tumor cells

**Fig. 8:**
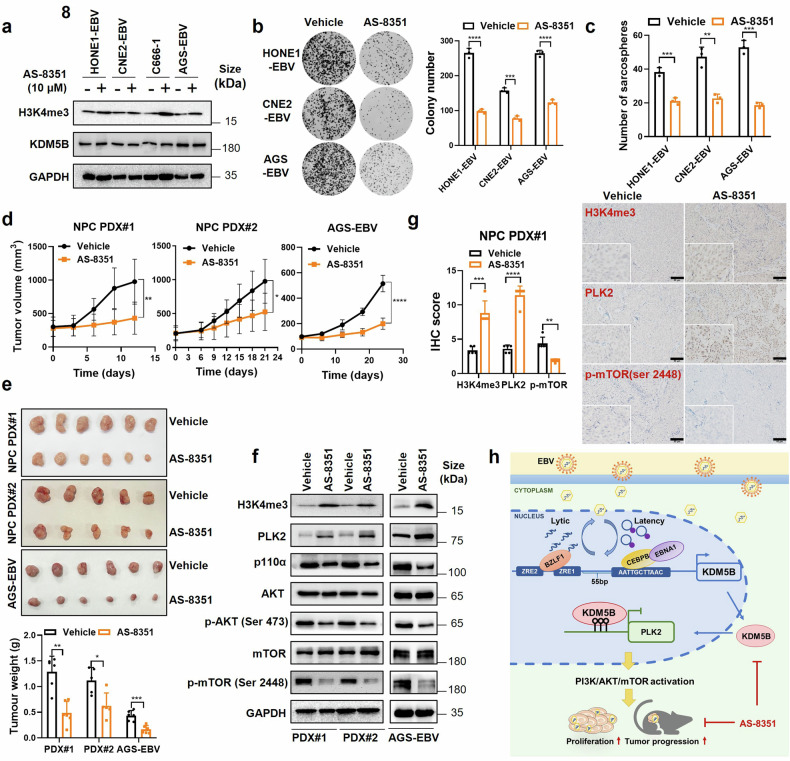
KDM5B inhibition reduces the malignancy of EBV-associated epithelial tumors. **a** Western blot assays detecting the protein levels of H3K4me3 and KDM5B in HONE1-EBV, CNE2-EBV, C666-1 and AGS-EBV cells treated with AS-8351 or vehicle control. GAPDH was used as a loading control. **b** Colony formation ability of HONE1-EBV, CNE2-EBV and AGS-EBV cells described in (**a**). The quantification of colony numbers is summarized at the right. **c** Sphere formation ability of HONE1-EBV, CNE2-EBV and AGS-EBV cells described in (**a**). **d** Tumor growth curves of NPC PDX model and AGS-EBV cells treated with AS-8351(25 mg/kg) or vehicle every two days. **e** Tumor size (upper) and weight (bottom) for the tumors excised from (**d**). **f** Western blot assays measuring the protein levels of H3K4me3, PLK2, p110α, AKT, p-AKT (Ser 473), mTOR, and p-mTOR (Ser 2448) in tumors from (**e**). GAPDH was used as a loading control. **g** Representative IHC staining images showing the protein levels of H3K4me3, PLK2, and p-mTOR (Ser 2448) in PDX tumors from (**e**). Statistical analysis of the staining results is presented on the left. **h** A hypothetical model illustrating how EBV-induced KDM5B expression facilitates the progression of EBV-associated epithelial tumors by activating the PLK2/PI3K/AKT/mTOR cascades. Statistical analysis is performed using Student’s *t-*test, with data presented as the mean ± SD. *P < 0.05, **P < 0.01, ***P < 0.001, ****P < 0.0001. SD, standard deviation. Scale bar, 50 μm

Considering the tumorigenic role of KDM5B, we explored the preclinical efficacy of KDM5B inhibition in EBV-associated epithelial tumors by applying a KDM5B small-molecule inhibitor, AS-8351, to EBV-positive epithelial tumor cells. Cell viability assay revealed that AS-8351 reduced tumor cell proliferation in a dose-dependent manner (Supplementary Fig. 10a). The IC50 values for AS-8351 treatment in HONE1-EBV, CNE2-EBV, and AGS-EBV cells were determined to be 21.13 μM, 40.53 μM, and 14.70 μM, respectively (Supplementary Fig. 10a). Based on these IC50 values, a concentration of 10 μM was selected as the optimal working concentration for subsequent treatments of these cell lines. Western blot analysis indicated that AS-8351 treatment upregulated H3K4me3 levels without altering *KDM5B* expression (Fig. 8a). Significantly, AS-8351 also reduced the proliferation, colony formation, and sphere formation abilities of these tumor cells (Fig. 8b, c and Supplementary Fig. 10b, c). Furthermore, treatment with AS-8351 in EBV-negative NPC and GC cells resulted in more modest inhibitory effects on cell proliferation compared to EBV-positive cells (Supplementary Fig. 10d, e). These findings support the potential of AS-8351 to inhibit tumor progression more effectively in EBV-positive tumor cells.

We next expanded our evaluation to in vivo settings, using xenografts from two EBV-positive NPC patient-derived xenografts (PDX; NPC PDX#1 and NPC PDX#2) and AGS-EBV cells. AS-8351 treatment led to a significant decrease in tumor growth in both NPC PDXs and AGS-EBV cells compared to vehicle-treated controls (Fig. 8d, e), without significant influence in body weight (Supplementary Fig. 10f). Additionally, we observed significantly decreased Ki-67 positive cells in the AS-8351 treated tumors (Supplementary Fig. 10g). Moreover, western blot assay revealed increase in H3K4me3 and PLK2 protein levels, concurrent with the inhibition of the PI3K/AKT/mTOR pathway, as evidenced by reduced expressions of p110 α, p-AKT (Ser 473), and p-mTOR (Ser 2448) in AS-8351 treatment groups compared to vehicle groups in NPC PDX models and AGS-EBV cell xenografts (Fig. 8f). IHC analysis corroborated these findings, showing elevated global levels of H3K4me3 and PLK2 expression, alongside with a reduction in p-mTOR levels following AS-8351 treatment (Fig. 8g and Supplementary Fig. 10h). These findings collectively highlight KDM5B inhibition, specifically through AS-8351, as a promising therapeutic strategy against EBV-associated epithelial tumors.

## Discussion

Accumulating evidence suggests a link between EBV infection and histone modifications, which result in altered chromatin accessibility and transcriptional dynamics.^16,31^ However, the precise molecular mechanisms underlying these changes have remained largely unknown. Our integrative analyses uncover for the first time that EBV infection leads to significant upregulation of *KDM5B* in EBV-associated epithelial cancers, with elevated levels correlating with poor patient survival. KDM5B is a well-known histone demethylase and master regulator of histone modifications that functions as a key mediator of chromatin organization, transcriptional control, and genome stability, all of which are critical for tumor progression across various malignancies.^25^ Consistently, our ChIP-seq analysis revealed substantial changes in KDM5B occupancy across promoters at genome-wide level upon manipulating *KDM5B* expression in NPC. These data suggest a link between EBV infection and KDM5B, mediating EBV-related malignant transformation.

Both latent infection and lytic replication of EBV have been implicated in the development of epithelial tumors.^5,6^ Our study reveals that EBNA1, a key latent viral protein consistently expresses across all EBV-associated cancers, collaborates with the transcription factor CEBPB at the *KDM5B* promoter to enhance its transcription. This interaction parallels previous findings in EBV-associated B-lymphoma cells, where EBNA1 forms a complex with Sp1 or Sp1-like proteins at the *Survivin* promoter to upregulate its expression.^32^ Besides this indirect transcriptional activation, EBNA1 can directly bind to motifs at the viral genome’s replication origin, activating other latency genes to facilitate latent infection.^33^ EBNA1 also binds to the promoters of host genes through its DNA-binding domain (DBD), notably upregulating *c-Jun* and *ATF2*, which are involved in NPC development and metastasis.^34^ In our study, we demonstrate that the DBD deletion still increased KDM5B expression, supporting our hypothesis that EBNA1 does not directly bind to the *KDM5B* promoter. Instead, EBNA1 likely recruits transcription factor CEBPB to modulate *KDM5B* expression. Furthermore, given that EBNA1 did not affect *KDM5B* expression in EBV-negative cells, we propose that other EBV-encoded factors may also play a role in this regulatory process. Additionally, EBNA1 has been reported to mediate the tethering of EBV and host genomes to regulate enhancers for NPC tumor progression.^31^ These findings elucidate the multifaceted capabilities of EBNA1 in coordinating EBV and host interactions, highlighting the importance of epigenetic modifications in the latent maintenance of EBV during cancer development.

Our study also demonstrates that EBV lytic induction increases *KDM5B* expression through the direct binding of BZLF1 to its promoter. BZLF1, an immediate-early gene of EBV encoding the Zta protein, functions as a transcriptional activator by recognizing Zta-response elements (ZREs) on viral gene promoters, thereby initiating the expression of EBV lytic genes.^4^ Beyond viral reactivation, BZLF1 has previously been shown to target and activate the transcription of host genes involved in immune modulation, such as *GM-CSF*, *IL-8*, and *IL-10*, in NPC.^35–37^ In this study, we establish that BZLF1 also targets the *KDM5B* promoter, providing a mechanistic link between EBV lytic activation and the dysregulation of host histone modifications. Furthermore, we found that inhibiting EBV DNA replication, which is essential for the expression of late lytic genes,^38^ does not impede the early lytic-induced upregulation of *KDM5B*. Given the well-established role of *KDM5B* in histone modifications,^25^ our findings highlight the robust capability of EBV lytic infection, even at incomplete (abortive) lytic cycle that does not progress to full DNA replication and virus production,^38^ to induce histone modifications and drive tumor progression in EBV-positive NPC and GC cells. Interestingly, although the ZREs for BZLF1 and the CEBPB binding motif are in close proximity within the *KDM5B* promoter, just 55 bp apart (Fig. 8h), our data demonstrates independent transcriptional activation of *KDM5B* by EBNA1 and BZLF1. This suggests that EBV strategically targets this critical region to exert precise control over the master regulator of histone modifications throughout both lytic and latent infection cycles.

Our study further delineates a functional link where KDM5B downregulates PLK2 expression by reducing the H3K4me3 levels at the promoter, thereby activating the PI3K/AKT/mTOR pathway to promote tumor progression in EBV-associated epithelial tumors. This is consistent with KDM5B’s tumor-promoting role through demethylation predominantly targeting histone 3 lysine 4 (H3K4) at promoters to transcriptionally silence the expression of tumor suppressor genes.^25,39^ Previous studies have demonstrated that KDM5B governs PI3K/AKT pathway activation through degrading PTEN in hepatocellular carcinoma^40^ and direct binding with *PIK3CA* promoter in prostate cancer.^30^ These findings indicate that KDM5B harnesses diverse regulatory mechanisms to activate PI3K/AKT pathway in tumor development, including epigenetic modifications of tumor suppressor genes like PLK2, degradation of PTEN, and direct transcriptional regulation of *PI3K*. Given that the PI3K/AKT pathway plays a crucial role in promoting proliferation, genomic instability, and reducing apoptosis in EBV-induced epithelial tumors,^28,41^ targeting KDM5B may be an effective strategy to suppress this downstream signaling cascade and combat EBV-associated epithelial tumors. Supportively, our study demonstrated that a small molecule inhibitor of KDM5B, AS-8351, effectively restrained the tumor progression of EBV-associated malignant cells in mice. Together with its relatively modest inhibitory effects on cell proliferation in EBV-negative cells, these findings underscore the therapeutic potential of AS-8351 in treating EBV-associated epithelial tumors.

In summary, our study reveals that EBV hijacks the host’s histone modification machinery during both latency and lytic phases of its life cycle, mediated by EBNA1 and BZLF1, respectively, to upregulate *KDM5B* expression. This EBV-driven upregulation of KDM5B promotes tumor progression by repressing the tumor suppressor PLK2 through H3K4me3 demethylation, thereby activating the PI3K/AKT/mTOR pathway (Fig. 8h). Our findings provide new insights into the sophisticated epigenetic mechanisms that EBV harnesses to drive tumor progression and suggest a promising therapeutic strategy targeting KDM5B and its molecular interactions for treating EBV-associated epithelial cancers. We acknowledge several limitations. First, given that KDM5B is a master histone modifier, additional downstream factors, beyond PLK2, may contribute to KDM5B-induced tumor progression in EBV-associated epithelial malignancies. Second, our integrative analysis revealed the upregulation of other histone modification enzymes, such as KDM4B and KDM5A, following EBV infection. Further exploration of these factors could provide insights into the broader role of histone modifications in EBV-mediated tumor progression, potentially identifying additional therapeutic targets for EBV-associated cancers.

## Materials and methods

### Ethics statement

This study adheres to all applicable ethical guidelines and regulations. This study was approved by the Institutional Review Boards at the Sun Yat-sen University Cancer Center (SYSUCC), Guangzhou, China. Written informed consent was obtained from all participants. Animal study (L025501202405011) was conducted with the approval from the institutional committee of SYSUCC and the procedures were performed in compliance with the ethical guidelines established by the Animal Experimentation Ethics Committee of SYSUCC.

### Clinical sample collection

Ten NPC samples for single-cell RNA sequencing (scRNA-seq) were collected between June 2018 and September 2018 at the SYSUCC as previously reported.^18^ For bulk RNA-seq, independent tumor biopsy samples from 113 NPC patients were retrieved from a previously published study.^42^ For immunohistochemistry (IHC) analysis, formalin-fixed paraffin-embedded (FFPE) biopsy specimens were obtained from NPC patients (*n* = 120) at the SYSUCC between June 2013 to March 2017. All samples were histopathologically confirmed as primary NPC in guideline with the World Health Organization (WHO) classification and EBV positivity was verified using in situ hybridization of EBV encoded small RNAs (EBERs). Tumor samples were collected before treatment. Clinical information for the samples is summarized in Supplementary Table 2.

### Cell culture and reagents

Human NPC cell lines (CNE2-EBV, HK1-EBV, S26, HONE1 and CNE2) and GC cell lines (AGS and AGS-EBV), and Burkitt lymphoma cell line Akata-EBV were kindly provided by Professor Mu-Seng Zeng from the SYSUCC. Human NPC cell line HONE1-EBV was kindly gifted by Professor Wei-Yi Fang from Southern Medical University (Guangzhou, China). Human NPC cell lines HK-1 and C666-1 were graciously shared by Professor KW Lo at the Chinese University of Hong Kong, Hong Kong SAR, China. Human embryonic 293T cells were sourced from the Cell Bank of Type Culture Collection of Chinese Academy of Sciences, Chinese Academy of Sciences. Recombinant EBV positive cell lines (CNE2-EBV, HK1-EBV, HONE1-EBV, AGS-EBV, Akata-EBV) were cultured in RPMI1640 medium supplemented with 10% fetal bovine serum (FBS), 1% penicillin–streptomycin solution (PS, Gibco, NY, USA) and G418 (700 μg/ml, Gibco, NY, USA). C666-1 and EBV negative cell lines (S26, HK-1 and AGS) were maintained in RPMI1640 medium supplemented with 10% fetal bovine serum (FBS) and 1% PS (Gibco, NY, USA). 293T cells were cultured in DMEM medium containing 10% FBS and 1% PS (Gibco, NY, USA). All cells were maintained at 37 °C with 5% CO_2_ in saturated humidity and were regularly confirmed to be free of mycoplasma contamination.

For the selection of cell lines, two NPC cell lines (HONE1-EBV and CNE2-EBV) and one GC cell line (AGS-EBV) were utilized for functional studies. HONE1-EBV, C666-1, and AGS-EBV were employed as model cell lines for mechanistic investigations. EBV-negative control cells included HK-1, S26, HONE1, CNE2, AGS, and non-malignant 293 T cell lines.

Primary antibodies for protein of interest were commercially available and manufactory information is provided in Supplementary Table 4; Secondary antibodies were HRP-linked anti-mouse IgG and anti-rabbit IgG, purchased from companies listed in Supplementary Table 4.

### EBV preparation and infection

EBV particles were generated using Akata-EBV cells as previously described.^43^ Briefly, Akata cells, which harbor GFP-tagged EBV, were suspended in serum-free RPMI 1640 medium at a concentration of 2 × 10^6^ cells/ml. These cells were then treated with 0.8% (v/v) goat anti-human IgG (Huayang Zhenglong Biochem Lab, Chengdu, China) at 37 °C for 6 h, after which the medium was replaced with RPMI1640 supplemented with 5% FBS. After culture for 72 h, viral particles in the supernatant were harvested under sterile conditions with a 0.45 μM Millipore filter, followed by high-speed centrifugation at 50,000 × *g*. The virus was then resuspended in fresh serum-free RPMI1640 at a volume of 1:100 of the original culture for concentration purposes. For EBV infection, EBV negative S26,^44^ HK-1,^45^ and AGS^46^ cells were plated in 24-well plates at a density of 4–6 × 10^4^ cells per well. Upon reaching approximately 60% confluence, the cells were washed with PBS twice and then infected with 200 ul of EBV suspension at 37 °C for a duration of 6 h. Afterwards, cells were again washed with PBS twice to eliminate unattached viral particles and maintained in fresh culture medium for 24–48 h. The EBV infection efficiency was assessed by identifying GFP-positive cells, using either fluorescence microscopy (Olympus, Tokyo, Japan) or flow cytometry (BD FACS Aria III, NY, USA).

### Transcriptome data generation and analyses

NPC scRNA-seq data was retrieved from a published study, which classified NPC cells into EBV-high and EBV-low cell clusters based on the expression of EBV-related molecules, including LMP-1, LMP-2A/B, RPMS1/A73, BNLF2a/b, and BNRF1.^18^ Differentially expressed genes between EBV-high and EBV-low tumor cells were identified utilizing the FindMarkers function within R package Seurat (version 4.1.1).^47^ Bulk RNA-seq data from tumor samples of NPC patients (*n* = 113) were collected from a previously published study (GSE102349), where RNA-seq libraries were generated using the Illumina TruSeq RNA Sample Preparation kit, which captures the full-length mRNA via oligo-dT.^42^ Additionally, bulk RNA-seq data from tumor cell lines were generated using standard procedures as previously described.^48^ Briefly, total RNA was extracted using the RNeasy Mini Kit (Qiagen, Germany). After removal of the ribosomal RNAs using Ribo‐Zero Magnetic Kit (Illumina, USA), library construction was conducted with TruSeq RNA prep kit (Illumina, USA). The libraries were then sequenced on the Illumina HiSeq XTen platform with a paired-end length of 150 bp. The sequencing data, provided in fastq format, were processed using RSEM and STAR software. High-quality pair-end reads were aligned using Bowtie 2. After filtering out ribosomal RNA, reads for each gene were quantified using HTseq. Gene expression levels were standardized to transcripts per million (TPM) or log2-transformed TPM (log2 TPM). Differential expression analysis was performed using DESeq2 software, with genes considered significantly differentially expressed if they had a log2 fold change greater than 1.5 and an adjusted *P*-value below 0.05. Spearman’s rank correlation test was employed to assess the correlations between the expression levels of KDM5B, transcription factors, and EBV-encoded genes.

### RNA extraction and RT-qPCR

Total cellular RNAs were isolated using Trizol reagents (Invitrogen, California, USA) according to the manufacturer’s protocols. Subsequently, the extracted RNA was reverse-transcribed into cDNA using the M-MLV Reverse transcriptase (Promega, Madison, WI, USA) and the oligo (dT) primers following the manufacturers’ instructions. Quantitative RT-PCR (RT-qPCR) was performed with the SYBR Premix Ex Taq kit (Takara, Tokyo, Japan). The specific primers employed for amplification were provided in Supplementary Table 5.

### siRNAs, plasmids construction, and lentivirus production

siRNAs targeting KDM5B, PLK2 and CEBPB (Gene Pharma, Shanghai, China) were introduced into cells with the Lipofectamine RNAiMAX reagent (Invitrogen, Carlsbad, CA, USA), in line with the manufacturer’s procedures. The expression vectors for BZLF1 (pcDNA3.0(+)-BZLF1) and BRLF1 (pEGFP-C1-BRLF1) were kindly gifted by Professor Mu-Seng Zeng from the SYSUCC. KDM5B (pLVX-Flag-KDM5B-Puro) and EBNA1 (pCXB-EBNA1) plasmids were purchased from Miao Ling Biology (Wuhan, China). Plasmids containing EBNA1’s DBD domain deletion mutants (EBAN1-DBD/GA-Dele) were constructed from pCXB-EBNA1 vector using mutation PCR. Full length cDNA of PLK2 was obtained using PCR and then cloned into the pCDH-CMV-MCS-EF1-Puro vector. shRNAs specifically targeting KDM5B (sh-KDM5B) were generated with the PLKO.1-puro vectors. For lentivirus production, target plasmids along with packaging plasmids psPAX2 and pMD2.G were co-transfected into 293 T cells following the manufacturer’s protocols. The cell culture media containing the resultant lentivirus was harvested to infect target cells, which were then maintained in a medium supplemented with puromycin (2 μg/mL). The siRNA and shRNA sequences are provided in Supplementary Table 5.

### Luciferase reporter assays

The promoters of KDM5B (spanning 2000 bp upstream of the TSS, designated as KDM5B-Luc) and PLK2 (encompassing 1500 bp upstream and 500 bp downstream distant to the TSS, referred to as PLK2-Luc) were obtained using PCR and cloned into the luciferase reporter pGL3-basic plasmid. Mutant reporter constructs with altered binding sites (CEBPBmt#1, CEBPBmt#2, JUNDmt#3, ZRE1mt, ZRE2mt, ZRE1/2mt) were generated from KDM5B-Luc vector using mutation PCR. Afterwards, these reporter plasmids along with pRL‐TK plasmid, were co-transfected into cells for 48 h. The luciferase activity was then quantified with the Dual‐Luciferase Reporter Assay system following the manufacturer’s procedures (Promega, Madison, USA, #E1910). The indicated primer sequences are listed in Supplementary Table 5.

### IHC and evaluation

IHC was performed as previously described.^48^ Briefly, formalin-fixed paraffin-embedded (FFPE) sections were initially dried at 60 °C for 1.5 to 2 h, then deparaffinized in xylene twice for 15 min each. Rehydration of the sections was achieved through successive immersion in graded ethanol solutions (100%, 95%, 80%, 70%) followed by distilled water. Subsequently, antigen retrieval was conducted by boiling the slides in sodium citrate solution. To block endogenous peroxidase activity, sections were treated with 3% hydrogen peroxide, followed by overnight incubation with primary antibodies at 4 °C. Afterward, secondary antibodies were applied at room temperature for 1 h, and DAB chromogenic staining was performed, followed by counterstaining with hematoxylin. IHC assessment was independently conducted by two pathologists at SYSUCC. Specifically, the IHC score was determined by multiplying the proportion of positively stained cells by the staining intensity (Supplementary Table 2), which was graded on a scale from 0 to 3: 0 for no staining, 1 for faint staining, 2 for moderate staining, and 3 for intense staining. Patients were grouped based on the median IHC score of KDM5B or PLK2 antibodies, with samples above the median classified as the high-expression group and those below the median as the low-expression group.

### Cell proliferation assays

For cell growth curves, 1 × 10^5^ cells were seeded in 12-well plates in triplicate. Cell numbers were quantified every 24 h over a three-day period utilizing a Cellometer Auto 1000 (Nexcelom Bioscience, Boston, USA). For colony formation assay, cells were plated in 6-well plates in triplicate at a density of 2 × 10^3^ per well and cultivated for 8–10 days at 37 °C in humidified incubator. Afterward, cells were fixed with 4% paraformaldehyde at room temperature for 20 min, then stained with a 0.5% crystal violet solution for 15 min. The stained colonies were imaged and analyzed using the Bio-Rad ChemiDoc Touch (Hercules, CA, USA).

### Cell viability assay

HONE1-EBV, CNE2-EBV, and AGS-EBV cells were seeded in 96-well plates at a density of 1 × 10³ cells/well and treated with various concentrations (0 μm, 2.5 μm, 5 μm, 10 μm, 15 μm, 20 μm and 50 μm) of AS-8351. After 24 h, cell viability was measured with the Cell Counting Kit-8 (CCK-8, Dojindo, Kyushu, Japan) according to the manufacturer’s protocols. At the designated time points, 10 µL of CCK-8 solution was added to each well, followed by a 2 h incubation. Absorbance was then assessed at 450 nm to evaluate cell proliferation. The half-maximal inhibitory concentration (IC50) values were calculated using nonlinear regression analysis of the cell viability data, performed with GraphPad Prism software (version 8.0).

### Sphere formation assays

1 × 10³ cells were seeded in six-well ultra-low attachment plates (Corning, NY, USA). The culture medium was DMEM/F12 (Gibco, NY, USA) supplemented with B27 medium (20 ng/ml, Gibco, NY, USA), human EGF (20 ng/ml, RD system, Minneapolis, USA) and human bFGF (20 ng/ml, RD system, Minneapolis, USA). After culture for 14 days, sarcospheres comprising more than 50 cells were quantitated using an inverted phase contrast microscopy (Olympus, Tokyo, Japan).

### Co-immunoprecipitation (co-IP) and western blot

For co-IP, cells were harvested and lysed in lysis buffer (Cell Signaling Technology, Danvers, USA) supplemented with 1× protease inhibitor cocktail (Topscience, shanghai, China) for 30 min. After centrifugation at 14,00 0× g for 10 min, protein concentration was determined, followed by incubation with specific antibodies at 4 °C for 4 h, and then with precleared protein A/G beads (Sigma, St. Louis, USA) overnight. The beads were subsequently washed with lysis buffer for five times. Proteins were eluted using 1×SDS buffer and boiled for 10 min, followed by subsequent western blot analysis. Briefly, protein samples underwent SDS-PAGE and were transferred to the PVDF membrane (Merck Millipore, Darmstadt, Germany). After blocking with 5% BSA (Sangon Biotech, Shanghai, China) in TBST buffer for 1 h, the membranes were then incubated with appropriate primary antibodies at 4 °C overnight, followed by incubation with secondary antibodies at room temperature for 1 h. Finally, immunoblotting was visualized using Fdbio-Dura ECL kit (Fdbio science, Hangzhou, China) and imaged with Bio-Rad ChemiDoc Touch system (Hercules, CA, USA).

### In vivo xenograft models

For the subcutaneous tumor model, four‐week‐old male BALB/c nude mice were purchased from Beijing Vital River Laboratory Animal Technology (Beijing, China) and maintained under specific pathogen-free conditions. The mice were randomly divided into different experimental groups. To initiate tumor growth, a mixture of 5 × 10^6^ CNE2-EBV or AGS-EBV cells with Matrigel (0.20 v/v, Corning, NY, USA) was prepared, resulting in a final volume of 100 µL per injection, and then subcutaneously injected into dorsal flank region of the nude mice. For Patient-derived xenograft (PDX) models, NPC PDX#1 was established using freshly acquired EBV-positive NPC tissue from SYSUCC. Small tissue samples were surgically transplanted into the abdomens of NSG mice. NPC PDX#2 was kindly provided by of Professor Cheng-Lung Hsu from Chang Gung University, Taiwan, China. Upon the tumors reaching a volume of approximately 100 mm³, the tumor-bearing mice received intraperitoneal injections bi-daily with AS-8351 (25 mg/kg), with vehicle as control. Tumor sizes were measured every 3 days. After 2–4 weeks, the mice were humanely euthanized, and the tumors were harvested and weighed. Tumor volume was calculated using the formula: volume = 0.52 × weight^2^ × length. All animal procedures were carried out following the regulations and ethical standards established by the Institutional Animal Care and Use Committee at SYSUCC.

### ChIP, ChIP-qPCR, and ChIP-seq analysis

For the ChIP assay, HONE1-EBV and AGS-EBV cells transfected with indicated expressing plasmids or control vectors were crosslinked with formaldehyde and subsequently processed using the Sonication ChIP Kit (#RK20258, ABclonal, Wuhan, China) according to the manufacturer’s protocols with EBNA1 or BZLF1 antibodies. ChIP-qPCR was performed using the SYBR Premix Ex Taq kit (Takara, Tokyo, Japan), and the primer sequences are provided in Supplementary Table 5. The enrichment of specific genomic regions was quantified relative to the input DNA and normalized to control IgG levels.

For ChIP-Seq, both ChIP and input DNAs were amplified and subjected to DNA library construction using Rapid Plus DNA Lib Prep Kit for Illumina V2 (Abclonal, Wuhan, China), following the manufacturer’s procedures Sequencing was performed on an Illumina HiSeq 2500 platform. ChIP-Seq data processing entailed the trimming of adapter sequences from both ends (10 bp) using Trim Galore (version 0.6.1). Subsequent alignment of the trimmed reads to the human reference genome hg38 was achieved using bowtie2 (version 2.5.0). Only mappings with a quality score above 10 were retained and organized using SAMtools (version 1.15), after which duplicates were removed utilizing the Picard toolkit (MarkDuplicates version 2.27.4). Peaks calling for KDM5B were conducted with MACS3 (version 3.0.0a7), excluding regions within ENCODE Blacklist areas.^49^ Peaks were annotated with ChIPseeker (version 1.32.1) and visualized using deepTools Toolkit (version 3.5.1). Promoter regions were identified as KDM5B peaks located within ±2 kb of any TSS as defined in refTSS (version 3.1).

### Statistical analysis

Statistical analysis was performed using GraphPad Prism version 8.0. The student’s t-test was employed for comparisons between two independent groups. One-way ANOVA followed by Tukey’s post hoc test (for comparisons among all groups), Sidak’s post hoc test (for comparisons between preselected groups), or Dunnett’s post hoc test (for comparisons with the control group) were used for comparisons involving more than two datasets. Pearson correlation analysis was utilized to examine the correlation between the expression of two genes. All experiments were replicated a minimum of three times, with results presented as mean ± standard deviation (SD). Survival curves were performed using the Kaplan–Meier method, with statistical significance calculated by the log-rank test. A *P*-value of less than 0.05 was considered statistically significant, with significance levels indicated as follows: * *P* < 0.05, ** *P* < 0.01, *** *P* < 0.001, **** *P* < 0.0001, and ns = nonsignificant.

## Supplementary information

Change of Authorship Request Form

Supplementary_Materials

## Data Availability

All relevant data are available upon request from the authors. Sequencing data involved in this article have been deposited in Genome Sequence Archive (GSA) with accession code HRA010229.^50^ Key raw data have been deposited in the Research Data Deposit (RDD) public platform (RDDB2025648402; http://www.researchdata.org.cn).
