# Supplementary material for: Epstein-Barr virus hijacks histone demethylase machinery to drive epithelial malignancy progression through KDM5B upregulation
Source: Signal Transduct Target Ther. 2025 Mar 10;10:83. doi: 10.1038/s41392-025-02163-5 (PMC11891327; doi:10.1038/s41392-025-02163-5)
Supplement: Supplementary file 1 — Change of Authorship Request Form [file 41392_2025_2163_MOESM1_ESM.pdf]

SPRINGER NATURE

Change of authorship request form - Journals (pre-acceptance)

Important information. Please read.

- This form should be used by authors to request any change in authorship (adding/deleting authors) including changes in corresponding authors. This form should not be used for name changes. Please fully complete all sections. Use black ink and block capitals and provide each author's full name with the given name first followed by the family name.

- By signing this declaration, all authors guarantee that the order of the authors are in accordance with their scientific contribution, if applicable as different conventions apply per discipline, and that only authors have been added who made a meaningful contribution to the work.

- Please note, in author collaborations where there is formal agreement for representing the collaboration, it is sufficient for the representative or legal guarantor (usually the corresponding author) to complete and sign the Authorship Change Form on behalf of all authors, **next to the added/removed author(s)**. **(Complete Section 3, followed by Section 6.)**
  In author collaborations where there is no formal agreement for representing the collaboration and **there are more than 10 authors**, one may sign for all, provided the signer appends correspondence that attests that each of the authors have agreed to the change **and the added/removed authors sign the form**. **(Complete Section 3, followed by Section 6.)**

- Please note, we cannot investigate or mediate any authorship disputes. If you are unable to obtain agreement from all authors (including those who you wish to be removed) you must refer the matter to your institution(s) for investigation. Please inform us if you need to do this.

- If you are not able to return a fully completed form within **30 days** of the date that it was sent to the author requesting the change, we may have to withdraw your manuscript. We cannot publish manuscripts where authorship has not been agreed by all authors (including those who have been removed).

- Incomplete forms will be rejected.

- Please return/upload this form, fully completed, to the Journals Editorial Office. The Journal and/or Publisher will consider the information you have provided to decide whether to approve the proposed change in authorship. We may decide to contact your institution for more information or undertake a further investigation, if appropriate, before making a final decision.

SPRINGER NATURE Change of authorship request form - Journals (pre-acceptance)

Section 1: Please provide the current title of manuscript

Manuscript ID no.: SIGTRANS-14883R1

Title: Epstein-Barr virus hijacks histone demethylase machinery to drive epithelial malignancy progression through KDM5B upregulation

Section 2: Please provide the previous authorship, in the order shown on the manuscript before the changes were introduced. Please indicate the corresponding author by adding (CA) behind the name.

	First name(s)	Family name	ORCID or SCOPUS id, if available
1st author	Ya-Qing	Zhou	
2nd author	Shuai	He	
3rd author	Yi-Qi	Li	
4th author	Xi-Xi	Cheng	
5th author	Jia-Xin	Jiang	
6th author	Shu-Qiang	Liu	
7th author	Pan-Pan	Wei	
8th author	Choon Kiat	Ong	
9th author	Vivien Ya-Fan	Wang	
10th author	Chun-Ling (CA)	Luo	

Please use an additional sheet if there are more than 10 authors.

	First name(s)	Family name	ORCID or SCOPUS id, if available
11th author	Jin-Xin (CA)	Bei	
12th author			
13th author			
14th author			
15th author			
16th author			
17th author			
18th author			
19th author			
20th author			

SPRINGER NATURE Springer Nature is one of the world's leading global research, educational and professional publishers, created in May 2015 through the combination of Nature Publishing Group, Palgrave Macmillan, Macmillan Education and Springer Science+Business Media.

SPRINGER NATURE # Change of authorship request form - Journals (pre-acceptance)

Section 3: Please provide a justification for change. Please use this section to explain your reasons for changing the authorship of your manuscript, e.g. what necessitated the change in authorship? Please refer to the (journal) policy pages for more information about authorship. Please explain why omitted authors were not originally included and/or why authors were removed on the submitted manuscript.

During the revision process, Professor Xin-Yuan Guan provided valuable guidance for the manuscript, while Jia-Xing Jiang significantly contributed to the experimental work. Consequently, we have added Professor Xin-Yuan Guan as a new author and revised Jia-Xing Jiang's position to second author.

Section 4: Proposed new authorship. Please provide your new authorship list in the order you would like it to appear on the manuscript. Please indicate the corresponding author by adding (CA) behind the name. <u>If the Corresponding Author has changed, please indicate the reason under section 3</u>.

	First name(s)	Family name (this name will appear in full on the final publication and will be searchable in various abstract and indexing databases)	Affiliated institute	E-mail address
1st author	Ya-Qing	Zhou	Sun Yat-sen University Cancer Center, Guangzhou, P. R. China.	zhouyq@sysucc.org.cn
2nd author	Jia-Xin	Jiang	Sun Yat-sen University Cancer Center, Guangzhou, P. R. China.	jiangjx@sysucc.org.cn
3rd author	Shuai	He	Sun Yat-sen University Cancer Center, Guangzhou, P. R. China.	heshuai@sysucc.org.cn
4th author	Yi-Qi	Li	Sun Yat-sen University Cancer Center, Guangzhou, P. R. China.	liyq2@sysucc.org.cn
5th author	Xi-Xi	Cheng	Sun Yat-sen University Cancer Center, Guangzhou, P. R. China.	chengxx@mail3.sysu.edu.cn
6th author	Shu-Qiang	Liu	Sun Yat-sen University Cancer Center, Guangzhou, P. R. China.	liusq@sysucc.org.cn
7th author	Pan-Pan	Wei	Sun Yat-sen University Cancer Center, Guangzhou, P. R. China.	weipp@sysucc.org.cn
8th author	Xin-Yuan	Guan	The University of Hong Kong, Hong Kong, China.	xyguan@hku.hk
9th author	Choon Kiat	Ong	National Cancer Centre Singapore, Singapore.	cmrock@nccs.com.sg
10th author	Vivien Ya-Fan	Wang	University of Macau, Taipa, Macau SAR, China.	vivienwang@um.edu.mo

Please use an additional sheet if there are more than 10 authors.

SPRINGER NATURE Springer Nature is one of the world's leading global research, educational and professional publishers, created in May 2015 through the combination of Nature Publishing Group, Palgrave Macmillan, Macmillan Education and Springer Science+Business Media.

SPRINGER NATURE

Change of authorship request form - Journals (pre-acceptance)

	First name(s)	Family name (this name will appear in full on the final publication and will be searchable in various abstract and indexing databases)	Affiliated institute	E-mail address
11th author	Chun-Ling (CA)	Luo	Sun Yat-sen University Cancer Center, Guangzhou, P. R. China.	luochl@sysucc.org.cn
12th author	Jin-Xin (CA)	Bei	Sun Yat-sen University Cancer Center, Guangzhou, P. R. China.	beijx@sysucc.org.cn
13th author				
14th author				
15th author				
16th author				
17th author				
18th author				
19th author				
20th author				

SPRINGER NATURE Springer Nature is one of the world's leading global research, educational and professional publishers, created in May 2015 through the combination of Nature Publishing Group, Palgrave Macmillan, Macmillan Education and Springer Science+Business Media.

Change of authorship request form - Journals (pre-acceptance)

Section 5: Author contribution, Acknowledgement and Disclosures. Please use this section to provide a new disclosure statement and, if appropriate, acknowledge any contributors who have been removed as authors and ensure you state what contribution any new authors made (if applicable per the journal or book (series) policy). Please ensure these are updated in your manuscript - after approval of the change(s) - as our production department will not transfer the information in this form to your manuscript.

New acknowledgements:

This work was supported by the National Key R&D Program of China (NKRDPC; 2021YFA1300201), the National Natural Science Foundation of China (NSFC; 82261160657, 81971270), the Guangdong Basic and Applied Basic Research Foundation (2024A1515013061), the Sci-Tech Project Foundation of Guangzhou City (2023A04J2141), Chang Jiang Scholars Program (J.-X.B.), the Hong Kong Research Grant Council (RGC) Theme-based Research Scheme Funds (T12-703/22-R and T12-703/23-N), the Science and Technology Development Fund, Macao S.A.R. (FDCT; 0089/2022/AFJ to V.Y.-F.W.), and National Medical Research Council (NMRC-OFLCG-23May0039 to C.K.O).

New Disclosures (financial and non-financial interests, funding):

We declare that we have no conflicts of interest.

New Author Contributions statement (if applicable per the journal policy):

Conceptualized and designed the study: J.X.B, C.L.L; procured financial support: J.X.B, C.L.L; sample preparation and data collection: Y.Q.Z, S.H, Y.Q.L, P.P.W; analyzed data: Y.Q.Z, S.H, Y.Q.L; performed functional experiments: Y.Q.Z, C.L.L, X.X.C, J.X.J, S.Q.L; result annotations: J.X.B, C.L.L, Y.Q.Z, C.K.O, V.Y.-F.W, X.Y.G; wrote the paper (original draft): Y.Q.Z, C.L.L, J.X.B. All authors revised and approved the manuscript.

State 'Not applicable' if there are no new authors

SPRINGER NATURE

Change of authorship request form - Journals (pre-acceptance)

Section 6: Declaration of agreement. *All* authors, unchanged, new and removed *must* sign this declaration.
(NB: Please print the form, (docu)-sign and return/upload a scanned copy. Please note that signatures that have been inserted as an image file are acceptable as long as it is handwritten. Typed names in the signature box are unacceptable.) *** Please delete as appropriate. Delete all of the bold if you were on the original authorship list and are remaining as an author.**

	First name	Family name		Signature	Date
1st author	Ya-Qing	Zhou	I agree to the proposed new authorship shown in section 4 and the proposed change in corresponding author	周雅清	Jan 27, 2025
2nd author	Jia-Xin	Jiang	I agree to the proposed new authorship shown in section 4 and the proposed change in corresponding author	[illegible]	Jan 27, 2025
3rd author	Shuai	He	I agree to the proposed new authorship shown in section 4 and the proposed change in corresponding author	何帅	Jan 27, 2025
4th authors	Yi-Qi	Li	I agree to the proposed new authorship shown in section 4 and the proposed change in corresponding author	[illegible]	Jan 27, 2025
5th author	Xi-Xi	Cheng	I agree to the proposed new authorship shown in section 4 and the proposed change in corresponding author	程茜茜	Jan 27, 2025
6th author	Shu-Qiang	Liu	I agree to the proposed new authorship shown in section 4 and the proposed change in corresponding author	刘树强	Jan 27, 2025
7th author	Pan-Pan	Wei	I agree to the proposed new authorship shown in section 4 and the proposed change in corresponding author	魏盼盼	Jan 27, 2025

SPRINGER NATURE Springer Nature is one of the world's leading global research, educational and professional publishers, created in May 2015 through the combination of Nature Publishing Group, Palgrave Macmillan, Macmillan Education and Springer Science+Business Media.

SPRINGER NATURE

Change of authorship request form - Journals (pre-acceptance)

	First name	Family name		Signature	Date
8th author	Xin-Yuan	Guan	I agree to the proposed new authorship shown in section 4 **and the addition of my name to the authorship list** and the proposed change in corresponding author		Jan 27, 2025
9th author	Choon Kiat	Ong	I agree to the proposed new authorship shown in section 4 and the proposed change in corresponding author		Jan 27, 2025
10th author	Vivien Ya-Fan	Wang	I agree to the proposed new authorship shown in section 4 and the proposed change in corresponding author		Jan 27, 2025

Please use an additional sheet if there are more than 10 authors.

	First name	Family name		Signature	Date
11th author	Chun-Ling (CA)	Luo	I agree to the proposed new authorship shown in section 4 and the proposed change in corresponding author		Jan 27, 2025
12th author	Jin-Xin (CA)	Bei	I agree to the proposed new authorship shown in section 4 and the proposed change in corresponding author		Jan 27, 2025

SPRINGER NATURE Springer Nature is one of the world's leading global research, educational and professional publishers, created in May 2015 through the combination of Nature Publishing Group, Palgrave Macmillan, Macmillan Education and Springer Science+Business Media.

SPRINGER NATURE

Change of authorship request form - Journals (pre-acceptance)

In case of author collaborations with formal agreement:

	Name of consortium/consortia	First name	Family name		Signature	Date
Representative/legal guarantor				I agree to the proposed new authorship shown in section 4 /**and the addition/removal*of my name to the authorship list** /and the proposed change in corresponding author		

Both added/removed authors should complete the information in the first table under Section 6.

---- End of form ----

SPRINGER NATURE Springer Nature is one of the world's leading global research, educational and professional publishers, created in May 2015 through the combination of Nature Publishing Group, Palgrave Macmillan, Macmillan Education and Springer Science+Business Media.
