# Supplementary material for: Epstein-Barr virus hijacks histone demethylase machinery to drive epithelial malignancy progression through KDM5B upregulation
Source: Signal Transduct Target Ther. 2025 Mar 10;10:83. doi: 10.1038/s41392-025-02163-5 (PMC11891327; doi:10.1038/s41392-025-02163-5)
Supplement: Supplementary file 2 — Supplementary_Materials [file 41392_2025_2163_MOESM2_ESM.pdf]

Supplementary Materials for

Epstein-Barr Virus Hijacks Histone Demethylase Machinery to Drive Tumor Progression in Epithelial Malignancies via KDM5B and PLK2 Interaction

Ya-Qing Zhou[1,2], Jia-Xin Jiang[1,3], Shuai He[1,3], Yi-Qi Li[1,3], Xi-Xi Cheng[1,3], Shu-Qiang Liu[1,3], Pan-Pan Wei[1,3], Xin-Yuan Guan[4], Choon Kiat Ong[5,6], Vivien Ya-Fan Wang[7], Chun-Ling Luo[1,3*], Jin-Xin Bei[1,3,8,9,10*]

Correspondence to: beijx@sysucc.org.cn (Bei JX) or luochl@sysucc.org.cn (Luo CL)

This PDF file includes:

Figures. S1 to S10

Tables S1 to S5

Other Supplementary Materials for this manuscript include the following:

Uncropped western blots

Supplementary Figures

Supplementary Figure 1

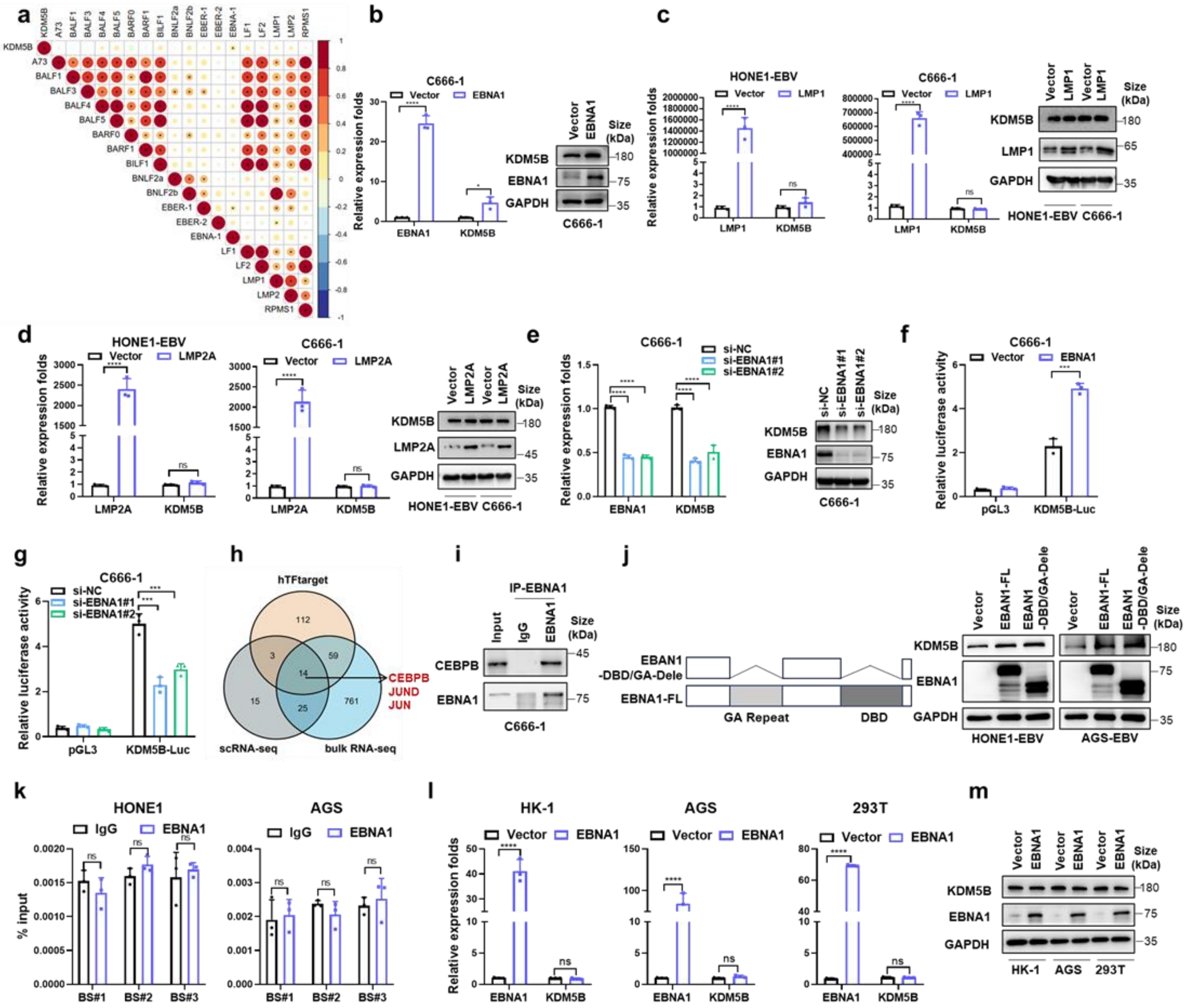

Supplementary Figure 1. EBNA1 interacts with CEBPB to promote *KDM5B* transcription. **a,** Correlation analysis between KDM5B expression and all EBV genes using in NPC bulk RNA-seq dataset (n=113). **b,** RT-qPCR (left) and western blot (right) analysis evaluating the expressions of EBNA1 and KDM5B in C666-1 cells transfected with *EBNA1* or empty vectors, with GAPDH serving as the loading control. **c,** RT-qPCR (left) and western blotting (right) analysis showing the expressions of LMP1 and KDM5B in HONE1-EBV and C666-1 cells transfected with either *LMP1* or empty vectors, with GAPDH serving as the loading control. **d,** RT-qPCR (left) and western blot (right) analysis evaluating the expressions of LMP2A and KDM5B in HONE1-EBV

and C666-1 cells transfected with either *LMP2A* or empty vectors, with GAPDH serving as the loading control. **e,** RT-qPCR (left) and western blot (right) analysis detecting expressions of EBNA1 and KDM5B in C666-1 cells transfected with siRNA (si-EBNA1#1 and si-EBNA1#2) or negative control (si-NC). **f-g,** Luciferase assay assessing the *KDM5B* transcription activity in C666-1 cells transfected with *KDM5B*-Luc, together with *EBNA1* overexpression vectors (**f**) or siRNAs (**g**). **h,** A Venn diagram illustrating the transcription factor numbers correlated with *KDM5B* expression, incorporating data from the hTFtarget database, EBV^{-}high cluster in NPC tissues (scRNA-seq; n=10), and bulk transcriptome data from NPC tumors (bulk RNA-seq; n=113). **i,** C666-1 cells were immunoprecipitated with EBNA1 antibody or immunoglobulin G (IgG), followed by western blot analysis with CEBPB and EBNA1 antibodies. **j,** Western blot analysis evaluating the protein levels of KDM5B and EBNA1 in HONE1-EBV and AGS-EBV cells transfected with EBNA1-FL, EBNA1-DBD/GA-Dele constructs, or control vectors. GAPDH was used as a loading control. The schematic diagram illustrating the design of the EBNA1-DBD/GA-deletion mutant, compared to the full-length EBNA1 (EBNA1-FL) presented left. **k,** ChIP-qPCR analysis for BS#1, BS#2 and BS#3 at the KDM5B promotor in wildtype EBV-negative HONE1 and AGS cells with EBNA1 or IgG antibodies. **l,** RT-qPCR assay quantifying the expression of EBNA1 and KDM5B in EBV-negative NPC (HK-1) and GC (AGS) cells, as well as non-malignant 293T cells, which were transfected with EBNA1-overexpressing or vector constructs. **m,** Western blot assay evaluating the protein levels of KDM5B and EBNA1 in cells described in (**l**), with GAPDH serving as a loading control. Statistical analysis is conducted using Student's t-test for two groups and one-way ANOVA followed by Dunnett's post hoc test for more than two groups. Data are presented as the mean ± SD. $^{*}P < 0.05$, $^{**}P < 0.01$, $^{***}P < 0.001$, $^{****}P < 0.0001$. ns, no significance. SD, standard deviation.

Supplementary Figure 2

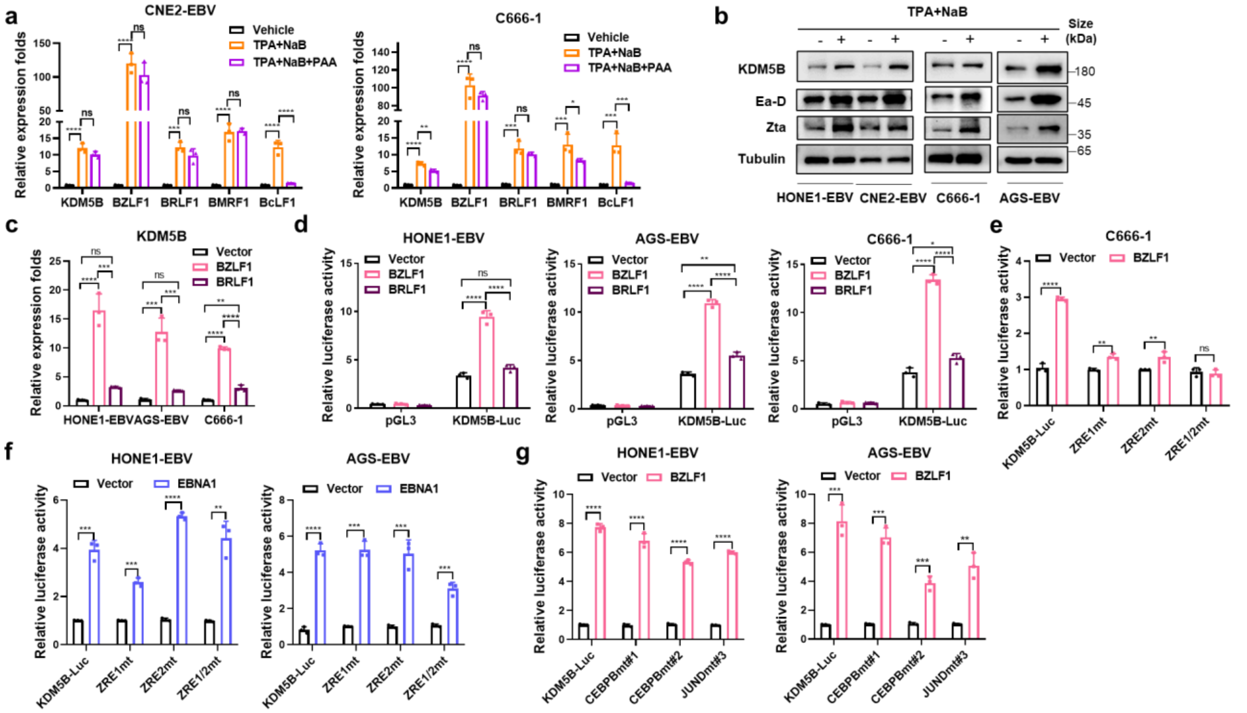

Supplementary Figure 2. BZLF1 binds to ZREs on *KDM5B* promoter to facilitate its transcription. a, RT-qPCR analysis detecting the expressions of EBV lytic genes (*BZLF1*, *BRLF1*, *BMRF1*, and *BcLF1*) and *KDM5B* in CNE2-EBV and C666-1 cells treated with TPA + NaB, TPA +NaB+PAA, or vehicle control. **b,** Western blot analysis assessing the protein levels of EA-D (BMRF1), Zta (BZLF1) and KDM5B in HONE1-EBV, CNE2-EBV, C666-1 and AGS-EBV cells treated either with or without TPA +NaB. Tubulin serves as a loading control. **c,** RT-qPCR analysis showing the *KDM5B* expression in HONE1-EBV, AGS-EBV and C666-1 cells transfected with either *BZLF1*, *BRLF1*, or empty vectors. **d,** Luciferase assays are performed to assess the *KDM5B* transcription activity in HONE1-EBV, AGS-EBV and C666-1 cells transfected with *KDM5B*-Luc, together with *BZLF1*, *BRLF1*, or control vectors. **e,** Luciferase assays to assess the *KDM5B* transcription activity in C666-1 cells transfected with *KDM5B*-Luc or mutant reporter plasmids corresponding to ZRE binding sites (labeled as ZRE1mt, ZRE2mt, ZRE1/2mt), alongside with *BZLF1* or control vectors. **f**, Luciferase assays evaluating the KDM5B transcription activity in HONE1-EBV and AGS-EBV cells transfected with either EBNA1 overexpression constructs or empty vector, alongside KDM5B-Luc or mutated BZLF1 binding site constructs (ZRE1mt, ZRE2mt, ZRE1/2mt). **g**, Luciferase assays evaluating the KDM5B transcription activity in

HONE1-EBV and AGS-EBV cells transfected with KDM5B-Luc or mutant reporter plasmids containing mutations of CEBPB or JUND binding motifs (CEBPBmt#1, CEBPBmt#2, JUNDmt#3), which concurrently overexpressing BZLF1 or control vector. Statistical analysis is conducted by Student's t-test for two groups and one-way ANOVA followed by Sidak's post hoc test for more than two groups. Data are presented as the mean ± SD. *$P < 0.05$, **$P < 0.01$, ***$P < 0.001$, ****$P < 0.0001$. ns, no significance. SD, standard deviation.

Supplementary Figure 3

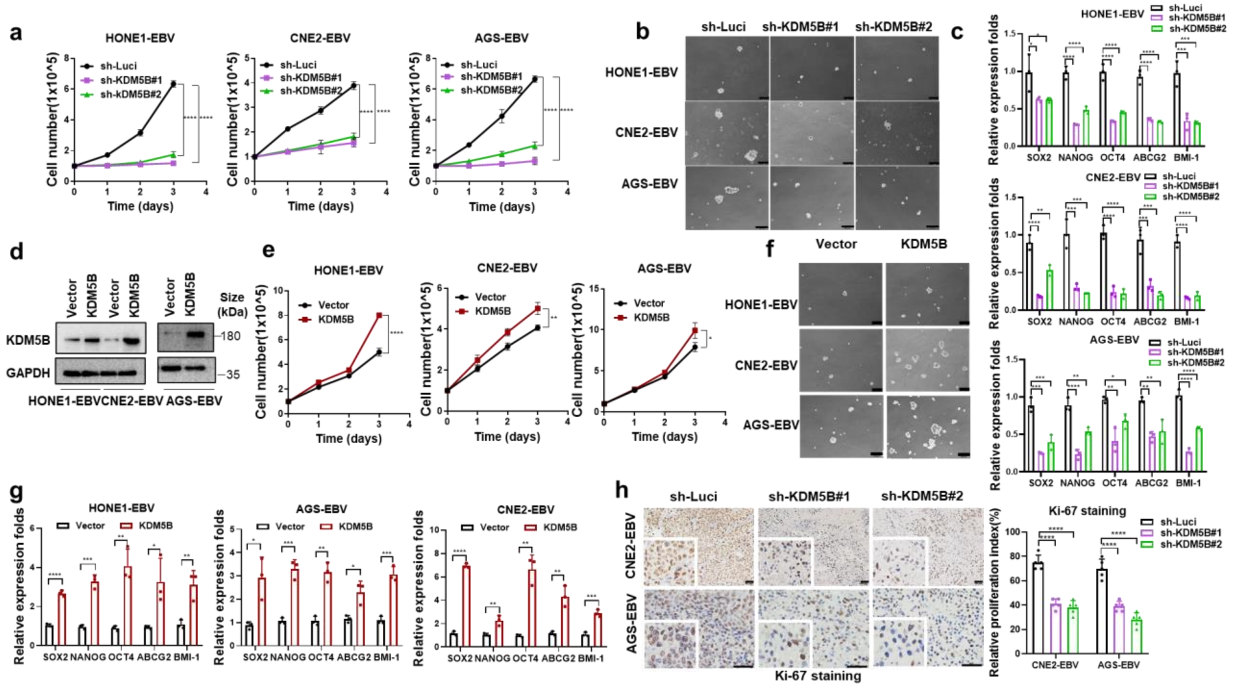

Supplementary Figure 3. KDM5B promotes tumor malignancy *in vitro* and *in vivo*. a, Cell growth curves of HONE1-EBV, CNE2-EBV and AGS-EBV cells infected with lentivirus expressing *KDM5B* shRNAs (sh-KDM5B#1 and sh-KDM5B#2) or control shRNA (sh-Luci). **b,** Sphere formation ability of cells described in (**a**), with representative images presented. Scale bar, 200 μm. **c,** RT-qPCR analysis evaluating the expression of stemness gene makers (*SOX2*, *NANOG*, *OCT4*, *ABCG2*, and *BMI-1*) in cells described in (**a**). **d,** Western blot assay showing the overexpression of KDM5B in HONE1-EBV, CNE2-EBV, and AGS-EBV cells infected with lentivirus expressing *KDM5B* or empty vector, using GAPDH as a control. **e,** Cell growth curves of HONE1-EBV, CNE2-EBV, and AGS-EBV cells infected with lentivirus expressing *KDM5B* or empty vectors. **f,** Sphere formation ability of cells described in (**e**), with representative images presented. Scale bar, 200 μm. **g,** RT-qPCR analysis evaluating the expression of stemness gene makers in cells described in (**e**). **h,** Representative images for IHC staining of Ki-67 in xenograft tumors from *KDM5B*-knockdown CNE2-EBV and AGS-EBV cells, with statistical analysis presented adjacent to the results. Scale bar, 50 μm. Statistical analysis is conducted using Student' s t-test for two groups and one-way ANOVA followed by Dunnett's post hoc test for more than two groups. Data are presented as the mean ± SD. *$P < 0.05$; **$P < 0.01$; ***$P < 0.001$, ****$P < 0.0001$. SD, standard deviation.

Supplementary Figure 4

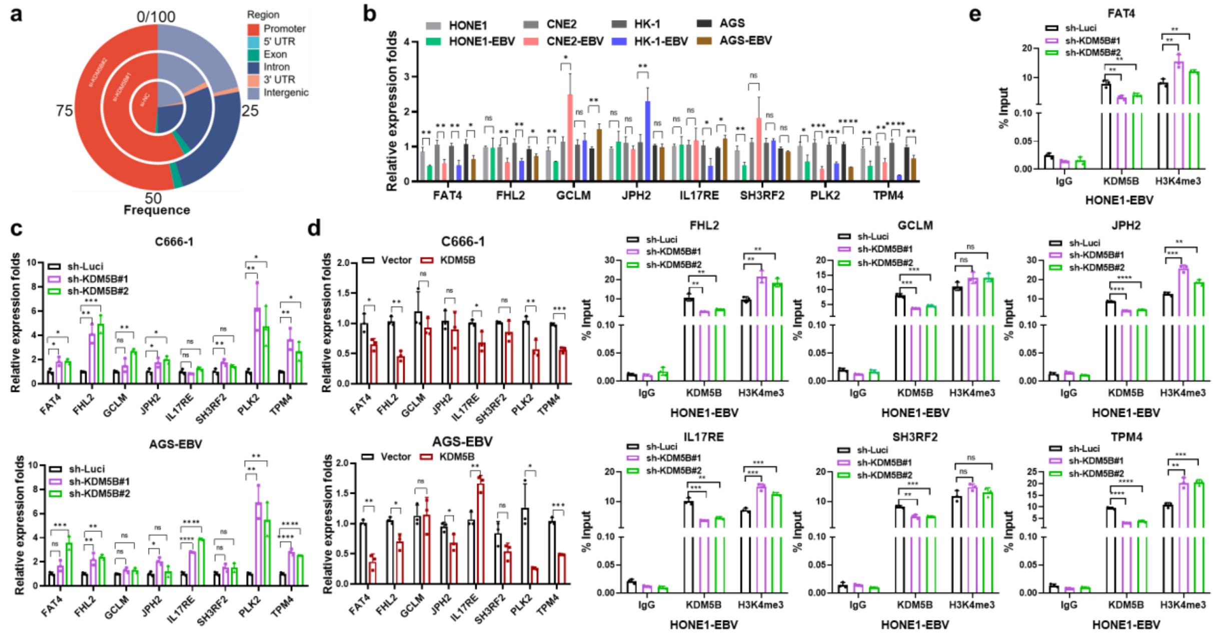

Supplementary Figure 4. Identification of PLK2 as the direct target of KDM5B. a, The Genomic distribution of KDM5B binding sites in HONE-EBV cells subjected to *KDM5B* knockdown (si-KDM5B#1 and si-KDM5B#2) compared to control (si-NC) cells. **b,** RT-qPCR analysis evaluating the expressions of potential *KDM5B*-regulated candidate genes (*FAT4*, *FHL2*, *GCLM*, *JPH2*, *IL17RE*, *SH3RF2*, *PLK2*, and *TPM4*) in NPC (HONE1, CNE2, and HK-1) and GC (AGS) cell lines with matched EBV-negative and EBV-positive pairs. **c-d,** RT-qPCR analysis assessing the expression of *FAT4*, *FHL2*, *GCLM*, *JPH2*, *IL17RE*, *SH3RF2*, *PLK2*, and *TPM4* in C666-1 and AGS-EBV cells with KDM5B knockdown (**c**) or overexpression (**d**). **e**, ChIP-qPCR analysis evaluating the binding of KDM5B and H3K4me3 at other potential KDM5B-regulated candidate genes (*FAT4*, *FHL2*, *GCLM*, *JPH2*, *IL17RE*, *SH3RF2* and *TPM4*) in HONE1-EBV cells with KDM5B knockdown by shRNAs. Statistical analysis is conducted using Student' s t-test for two groups and one-way ANOVA followed by Dunnett's post hoc test for more than two groups. Data are presented as the mean ± SD. *, $P < 0.05$, **$P < 0.01$, ***$P < 0.001$, ****$P < 0.0001$. ns, no significance. SD, standard deviation.

Supplementary Figure 5

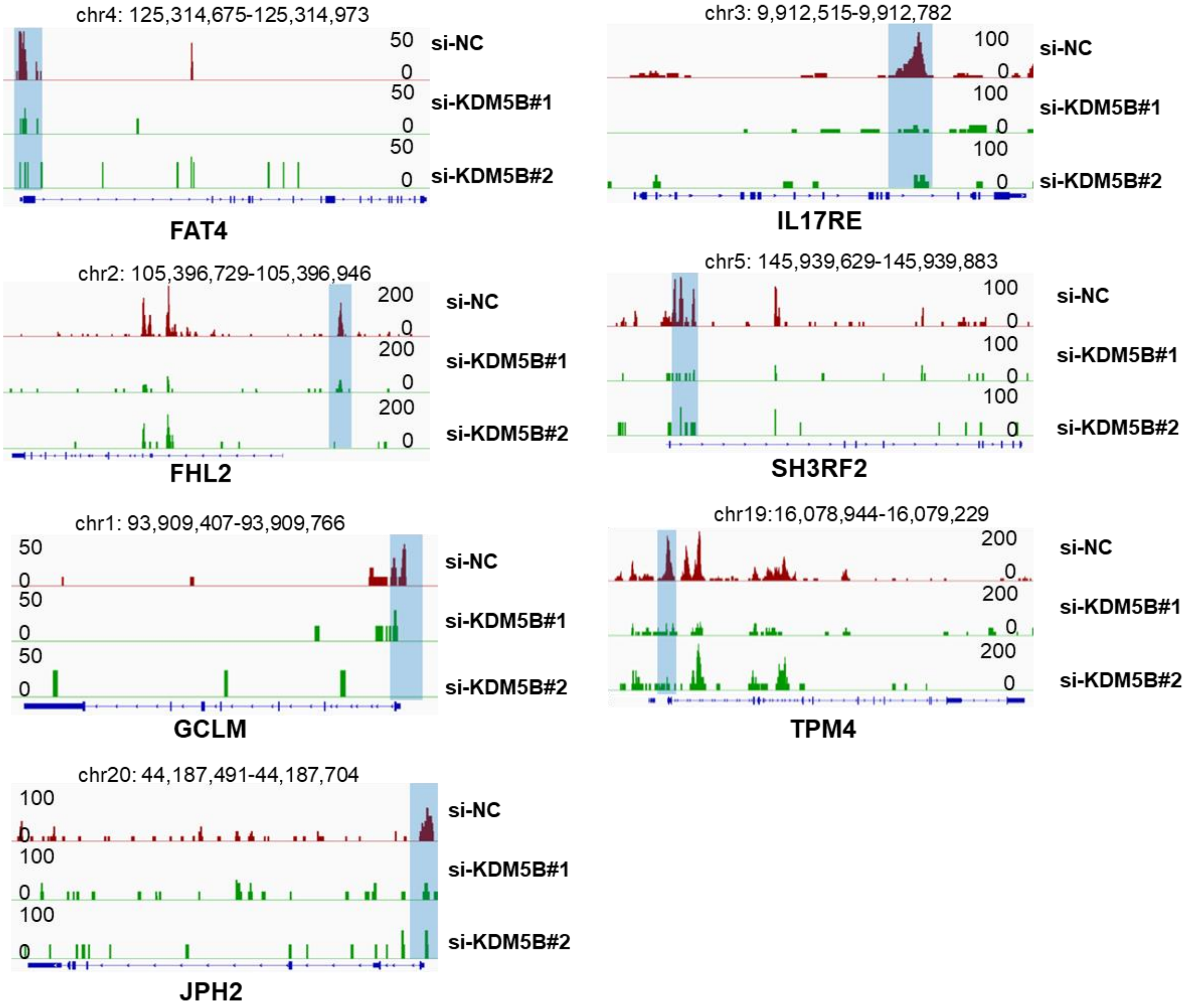

Supplementary Figure 5. ChIP-seq analysis of KDM5B signals in its target genes. ChIP-seq profiling KDM5B occupying signals in FAT4, FHL2, GCLM, JPH2, IL17RE, SH3RF2 and TPM4 loci following KDM5B knockdown by siRNAs (#1/2).

Supplementary Figure 6

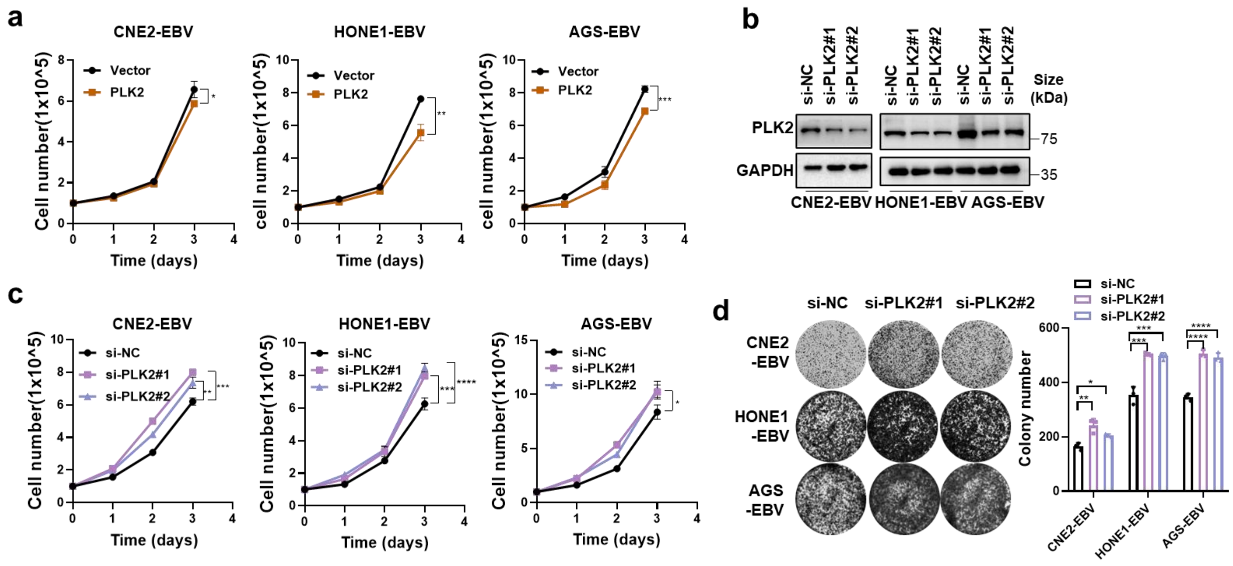

Supplementary Figure 6. PLK2 act as a suppresser in EBV-positive epithelial tumor cells. a, Cell growth assay of CNE2-EBV, HONE1-EBV and AGS-EBV cells infected with lentivirus expressing *PLK2* or empty vector. **b,** Western blot assay to detect the protein level of PLK2 in CNE2-EBV, HONE1-EBV, and AGS-EBV cells transfected with siRNAs (si-PLK2#1 and si-PLK2#2) or negative control (si-NC). GAPDH serves as a loading control. **c-d,** Cell growth (**c**) and colony formation (**d**) assays results for cells described in (**b**).

Supplementary Figure 7

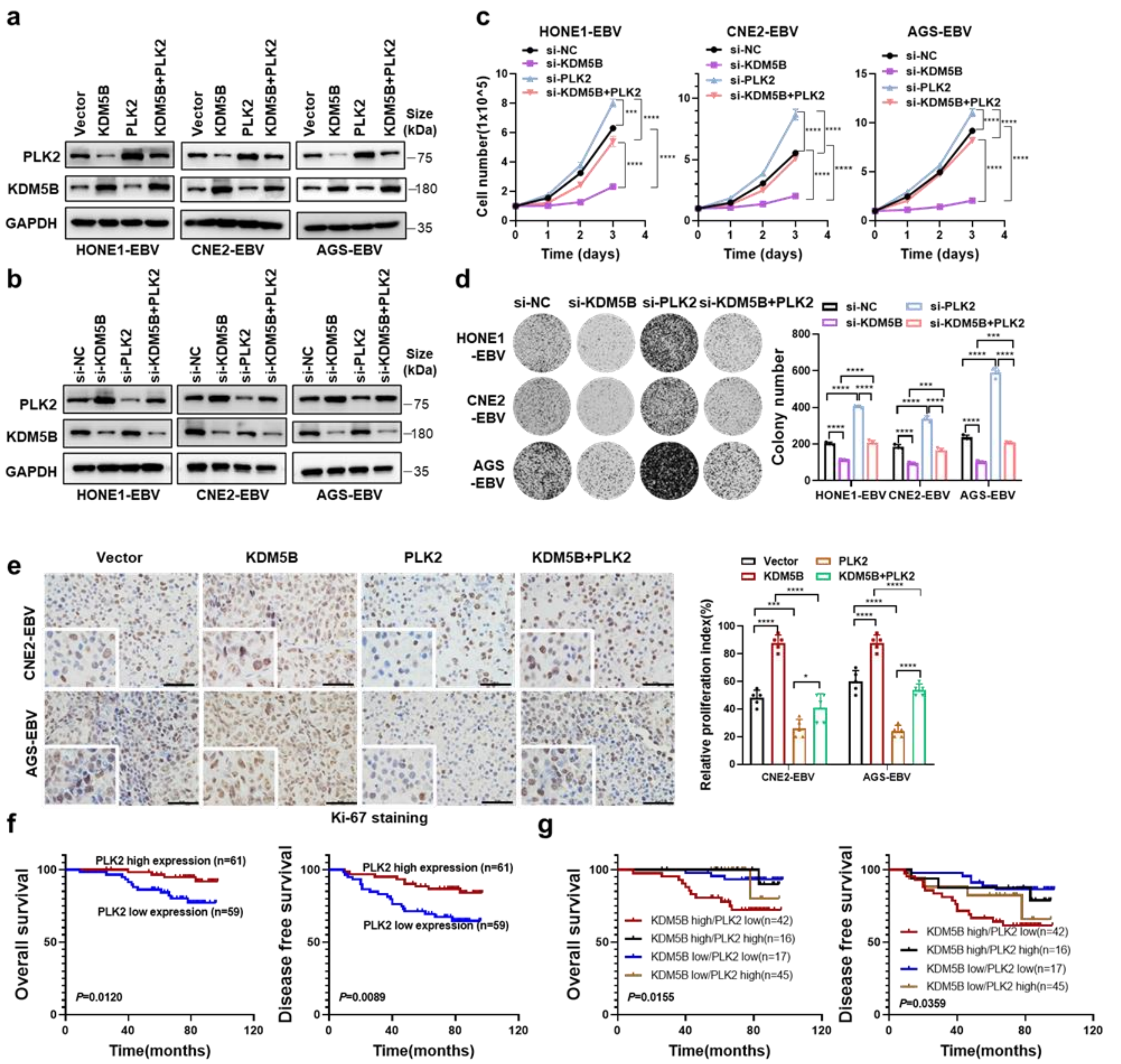

Supplementary Figure 7. PLK2 contributes to KDM5B-induced proliferation in EBV-positive epithelial tumor cells. a, Western blot assay measuring the protein level of KDM5B and PLK2 in matched EBV- positive NPC (HONE1-EBV, CNE2-EBV) and GC (AGS-EBV) cells stably overexpressing KDM5B or PLK2 alone, or concurrently overexpressing KDM5B and PLK2. using GAPDH as a control. **b,** Western blot assay detecting the protein level of KDM5B and PLK2 in HONE1-EBV, CNE2-EBV and AGS-EBV cells transfected with control siRNA, KDM5B siRNA, PLK2 siRNA, or simultaneous KDM5B and PLK2 siRNAs, using GAPDH as a control.

c, Cell growth curves of cells described in (**b**). **d**, Colony formation ability of cells described in (**b**), with statistics presented at the right. **e,** Representative images for IHC staining of Ki-67 in xenograft tumors from CNE2-EBV and AGS-EBV cells stably overexpressing KDM5B or not (control vector), followed by infection of lentivirus expressing PLK2 or empty vector, with statistical analysis presented on the right. **f,** Kaplan-Meier survival curves of overall and disease-free survival stratified by PLK2 expression according to IHC staining in NPC patients (n=120). **g,** Kaplan-Meier survival curves of overall and disease-free survival stratified by both PLK2 and KDM5B expression in NPC patients detailed in (**f**). Statistical analysis is conducted using Student's t-test for two groups and one-way ANOVA followed by Dunnett's post hoc test or Sidak's post hoc test for more than two groups. Data are presented as the mean ± SD. *$P < 0.05$, ** $P < 0.01$, ***$P < 0.001$, ****$P < 0.0001$. SD, standard deviation. Scale bar, 50 μm.

Supplementary Figure 8

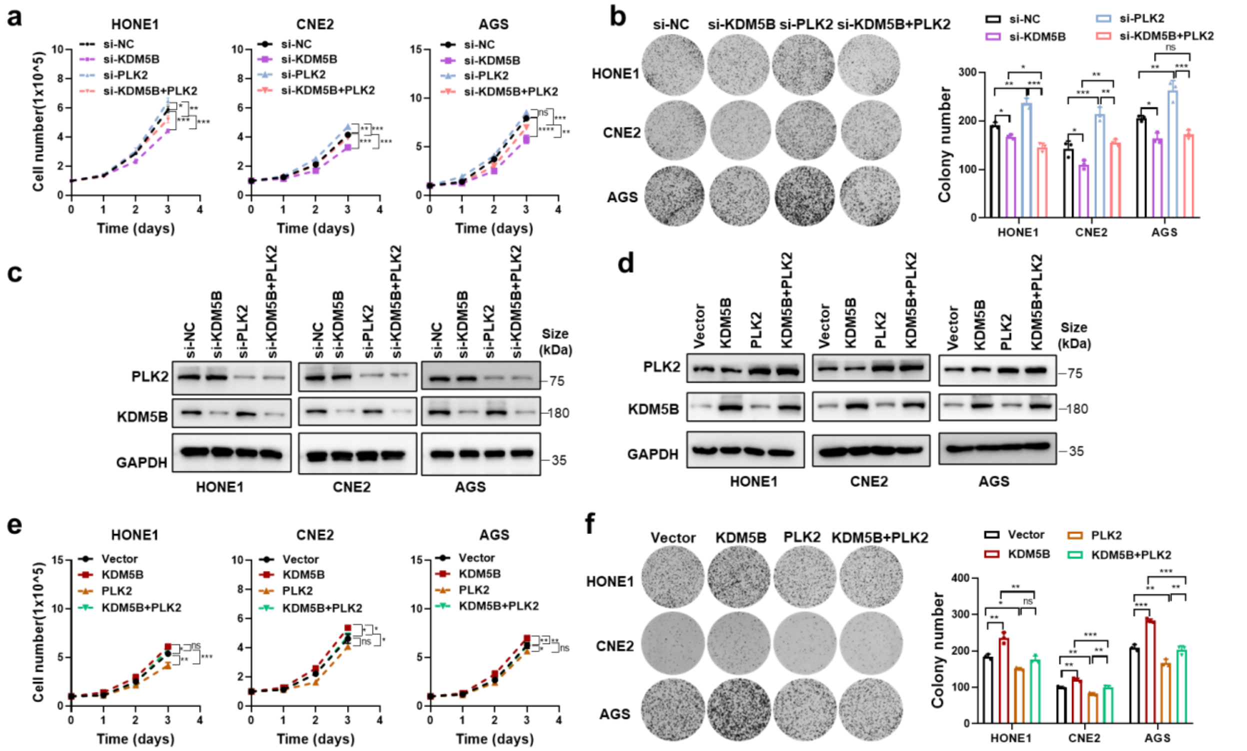

Supplementary Figure 8. KDM5B/PLK2 axis plays a weak role in EBV-negative cells. a, Cell growth curves of EBV-negative NPC (HONE1, CNE2) and GC (AGS) cells transfected with control siRNA, KDM5B siRNA, PLK2 siRNA, or simultaneous KDM5B and PLK2 siRNAs. **b**, Colony formation ability of cells described in (**a**), with statistics presented at the right. **c**, Western blot assay detecting the protein level of KDM5B and PLK2 in cells described in (**a**), using GAPDH as a control. **d,** Western blot assay measuring the protein level of KDM5B and PLK2 in EBV-negative NPC (HONE1, CNE2) and GC (AGS) cells stably overexpressing KDM5B or PLK2 alone, or concurrently overexpressing KDM5B and PLK2, using GAPDH as a control. **e**, Cell growth curves of cells described in (**d**). **f**, Colony formation ability of cells described in (**d**), with statistics presented at right. Statistical analysis is conducted using Student's t-test for two groups and one-way ANOVA followed by Dunnett's post hoc test or Sidak's post hoc test for more than two groups. Data are presented as the mean ± SD. *$P < 0.05$, ** $P < 0.01$, ***$P < 0.001$, ****$P < 0.0001$. ns, no significance. SD, standard deviation.

Supplementary Figure 9

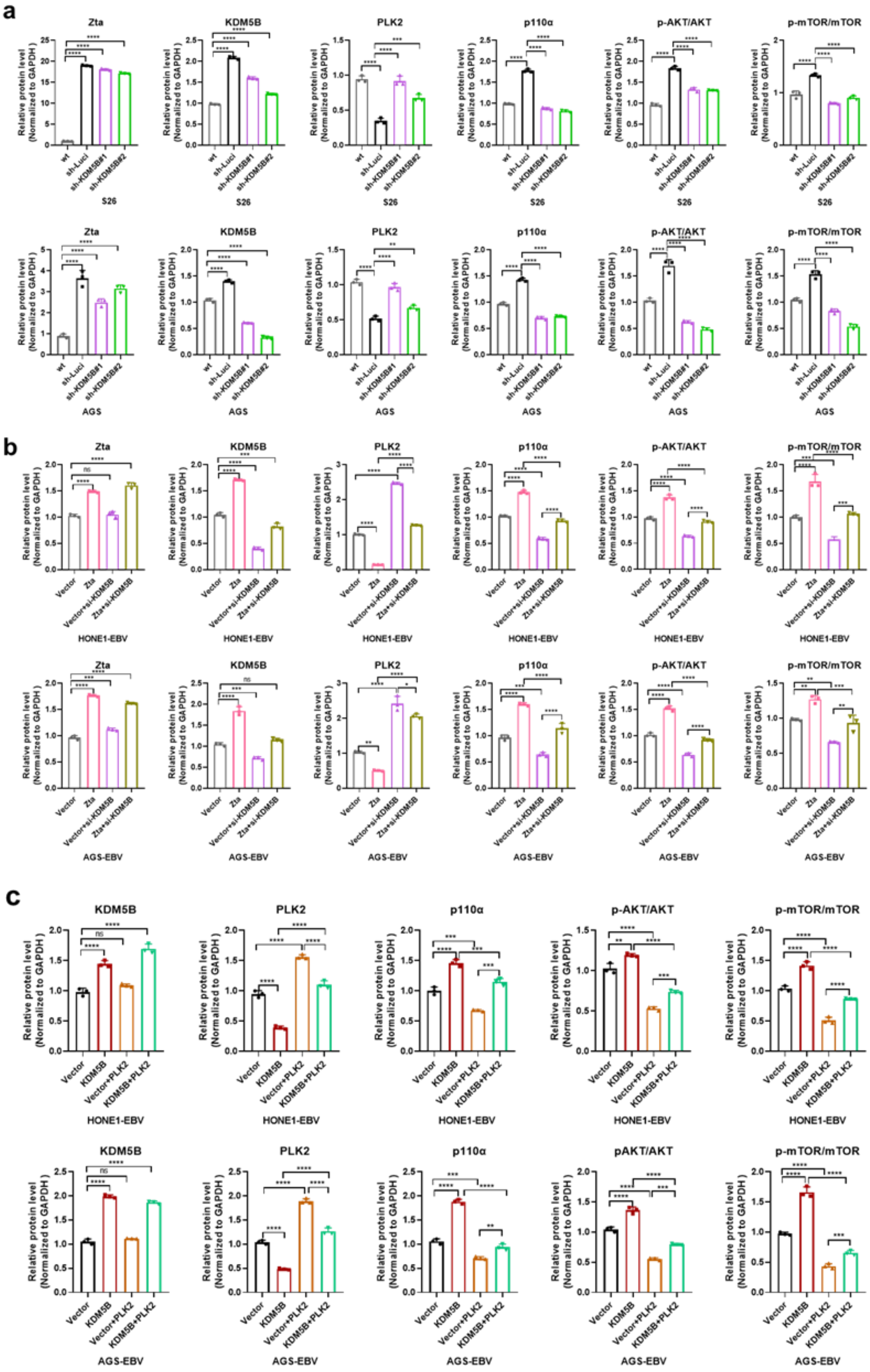

Supplementary Figure 9. Quantification of western blot results in Figure 7. a, Quantified protein levels of Zta, KDM5B, PLK2, p110α, p-AKT (Ser 473)/AKT, p-mTOR (Ser 2448)/mTOR in parental or EBV-infected S26 and AGS cells together with KDM5B knockdown by shRNAs or not. Quantified results were normalized to GAPDH. **b**, Quantified protein levels of the indicated proteins in HONE1-EBV and AGS-EBV cells, transfected with either Zta or empty vector, followed by subsequent transfection of siRNA targeting KDM5B. Quantified results were normalized to GAPDH. **c**, Quantified expressions of indicated proteins in HONE1-EBV and AGS-EBV cells with stable overexpression KDM5B or an empty vector, followed by infection with lentivirus overexpressing PLK2. Quantified results were normalized to GAPDH. Statistical analysis is conducted using one-way ANOVA followed by Sidak's post hoc test. Data are presented as the mean ± SD. *$P < 0.05$, ** $P < 0.01$, ***$P < 0.001$, ****$P < 0.0001$. SD, standard deviation.

Supplementary Figure 10

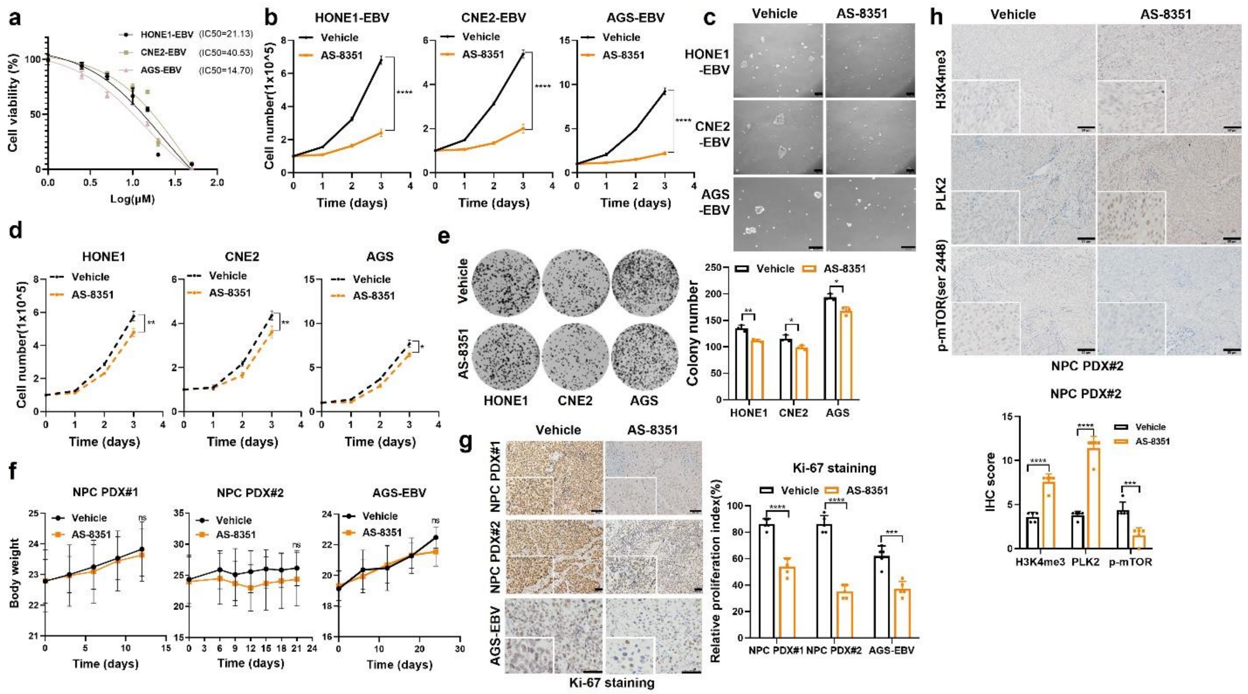

Supplementary Figure 10. KDM5B inhibition decreases tumor growth *in vitro* and *in vivo*. a, Cell viability of HONE1-EBV, CNE2-EBV and AGS-EBV cells treated with AS-8351 at different concentrations (2.5 μM, 5 μM, 10 μM, 15 μM, 20 μM and 50 μM) or control vehicle, and the IC50 values are shown alongside. **b,** Cell growth curves of HONE1-EBV, CNE2-EBV and AGS-EBV cells treated with AS-8351(10 μM) or vehicle. **c,** Sphere formation ability of cells described in (**b**), with representative images presented. **d**, Cell growth curve of EBV-negative NPC (HONE1, CNE2) and GC (AGS) cells treated with AS-8351(10 μM) or vehicle control. **e**, Colony formation ability of cells described in (**d**). The quantification of colony numbers is summarized at the right. **f,** The body weight curves of mice bearing NPC PDXs (NPC PDX#1 and NPC PDX#2) or AGS-EBV cell xenografts, which are treated with AS-8351 (25 mg/kg) or vehicle. **g,** Representative images for IHC staining of Ki-67 in NPC PDXs and AGS-EBV cell xenograft described in (**f**). Statistical analysis of the staining results is presented at right. **h,** Representative IHC staining images showing the protein levels of H3K4me3, PLK2, and p-mTOR (Ser 2448) in PDX tumors from (**f**). Statistical analysis of the staining results is presented below. Statistical analysis is conducted using Student'

s t-test and data are presented as the mean ± SD. $*P < 0.05$, $**\ P < 0.01$, $***P < 0.001$, $****P < 0.0001$. ns, no significance. SD, standard deviation. Scale bar, 50 μm.

Supplementary figure 11

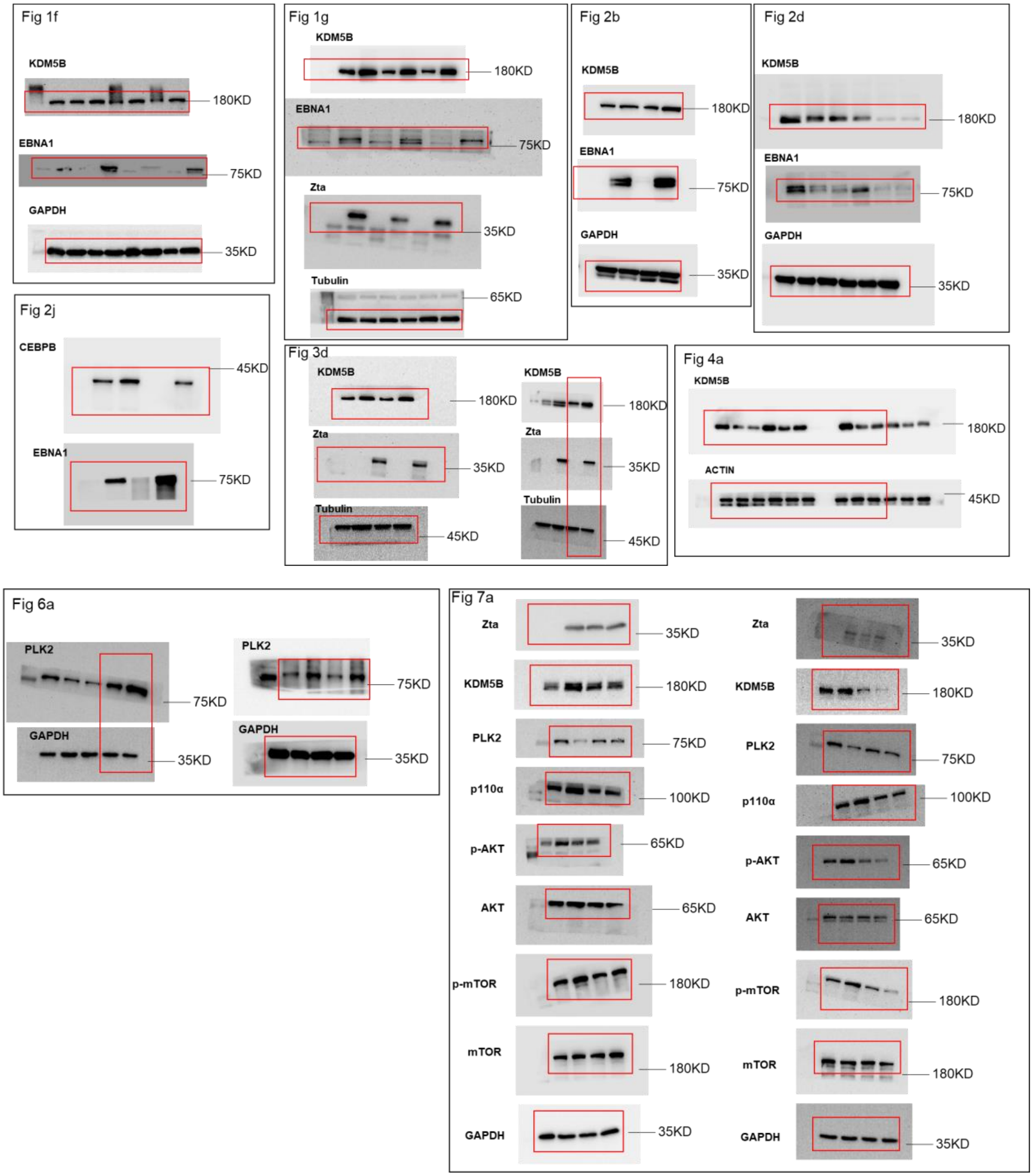

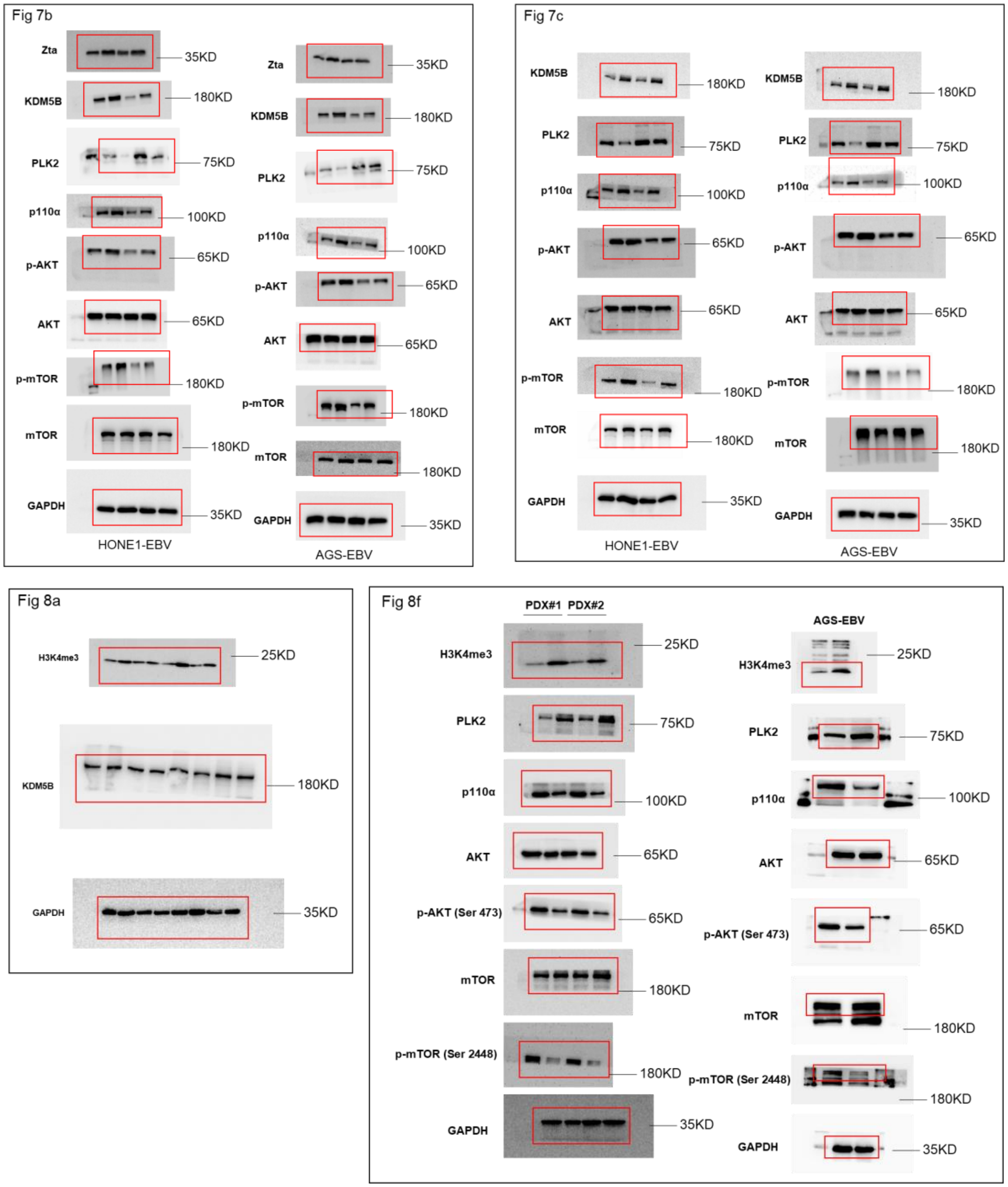

Supplementary figure 11. Uncropped western blots for Figures 1-8.

Supplementary figure 12

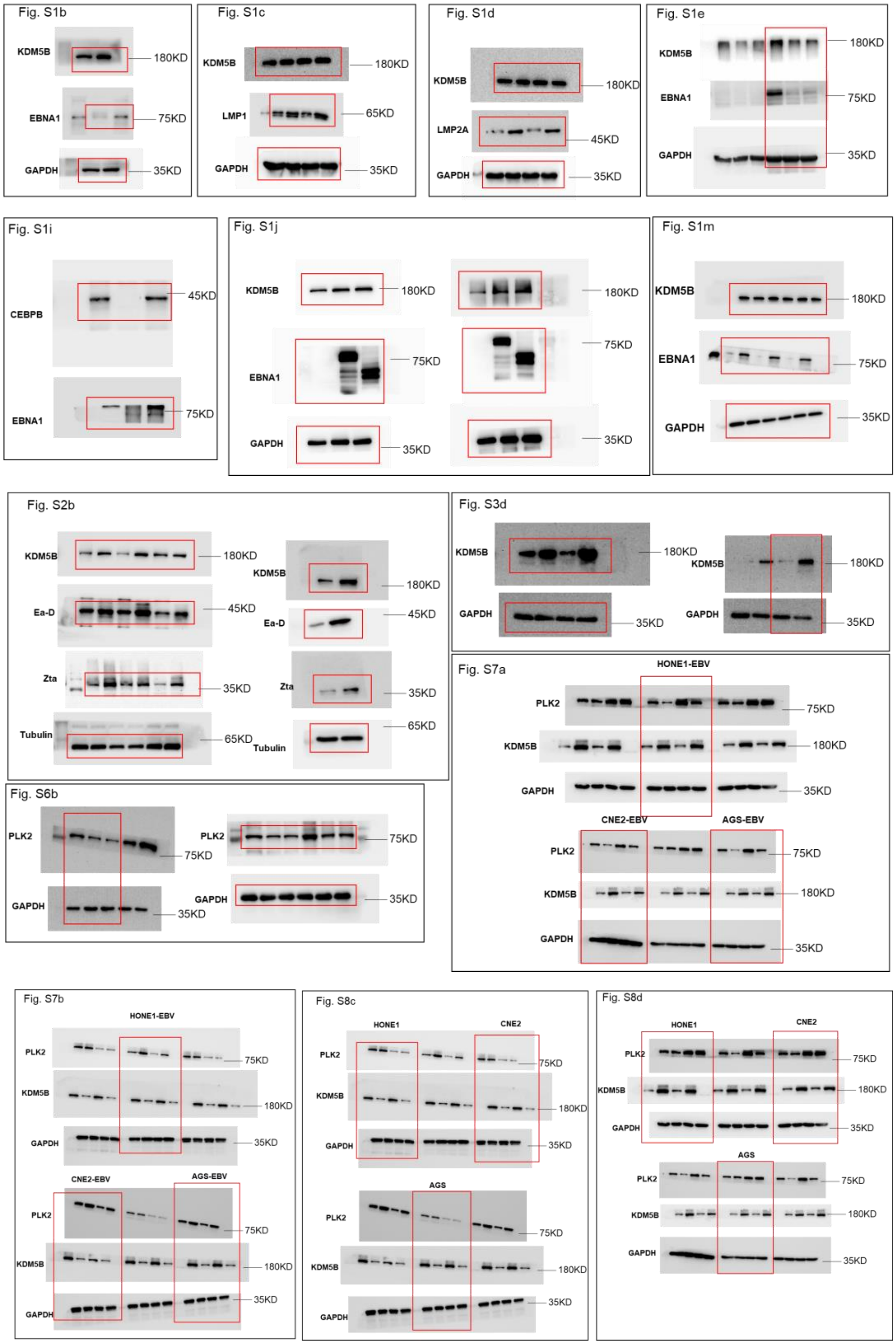

Supplementary figure 12. Uncropped western blots for Supplementary figures 1-10.

Supplementary Table 1. Upregulated histone modifiers in NPC and GC cells upon EBV infection.

Samples	Number	Gene	avg_logFC	p_val_adj
EBV^{high} and EBV^{low} malignant epithelial cells from 10 NPC tissues	1	KDM5A	0.48439	1.03E-131
	2	KDM5B	0.4227982	1.56E-125
	3	WHSC1L1	0.3533266	1.93E-110
	4	KDM2A	0.3192056	3.62E-105
	5	ASH1L	0.3102985	2.94E-98
	6	USP22	0.2845795	8.01E-82
	7	GSK3B	0.2629588	1.17E-86
	8	KDM6B	0.2612672	1.98E-74
	9	HUWE1	0.2567563	7.83E-85
	10	PRKDC	0.2542981	3.67E-56
	11	MGEA5	0.2413334	7.46E-73
	12	KDM6A	0.2351667	1.36E-66
	13	MAP3K8	0.2294961	8.26E-59
	14	KDM1A	0.1963744	1.66E-62
	15	PRDM2	0.1824534	1.56E-65
	16	BAZ1B	0.1784146	2.97E-58
	17	CDK17	0.1732682	6.19E-60
	18	UBR2	0.1681201	1.16E-52
	19	KDM2B	0.1625176	7.99E-45
	20	USP16	0.1613458	8.17E-46
	21	EP300	0.1602403	4.58E-54
	22	NCOA3	0.1535359	2.98E-42
	23	SETD2	0.1496468	9.56E-49
	24	STK4	0.1466339	1.13E-51
	25	LIMK2	0.1457728	4.34E-43
	26	KDM4A	0.1447172	9.63E-56
	27	JAK2	0.1432578	8.78E-37
	28	ATR	0.1378876	2.67E-41
	29	TLK1	0.1352454	5.28E-52
	30	GTF3C4	0.1304247	1.46E-52
	31	EHMT1	0.1250926	4.64E-40
	32	CREBBP	0.1238823	3.03E-38
	33	KDM4B	0.1230901	1.25E-34
	34	MYSM1	0.1228509	2.46E-39
	35	USP3	0.1215611	3.18E-48
	36	NSD1	0.1190825	9.40E-51
	37	SIRT1	0.1172638	1.16E-37
	38	KDM3B	0.1152057	3.63E-47
	39	RNF168	0.1149926	1.80E-39
	40	NAT10	0.1077567	8.59E-37
	1	KDM5B	1.165676	6.58E-10

S26 cells with EBV infection *in vitro*	2	HDAC5	0.916972	3.41E-07
	3	HDAC11	0.867562	0.044131
	4	KDM4B	0.768059	0.000154
	5	SIRT2	0.745829	0.000542
	6	PRMT2	0.718006	0.001699
	7	HDAC6	0.591937	0.007041
	8	CDK5	0.535505	0.047734
	9	SIRT6	0.441237	0.026071
	10	KDM5C	0.367805	0.034933
	11	EHMT2	0.346649	0.024661
HK-1 with EBV infection *in vitro*	1	HDAC9	1.02426	2.46E-25
	2	KDM6B	0.877675	4.79E-28
	3	KDM5B	0.839006	1.18E-29
	4	CDK17	0.731154	2.01E-08
	5	KDM5C	0.611101	2.72E-22
	6	CDYL	0.587396	5.04E-07
	7	HDAC5	0.55776	2.90E-13
	8	PHF8	0.550021	2.08E-06
	9	NCOA3	0.527388	5.75E-16
	10	RAG1	0.506028	0.001244
	11	KDM4B	0.47642	4.78E-05
	12	SIRT1	0.455086	0.001795
	13	RPS6KA5	0.425038	0.007848
	14	KDM1B	0.39898	0.001465
	15	SETD1B	0.368903	0.000611
	16	GSK3B	0.360629	1.28E-07
	17	KDM4A	0.354955	1.06E-05
	18	TLK1	0.318999	0.001912
	19	SIRT6	0.272906	0.010013
	20	HUWE1	0.265365	8.83E-10
	21	CLOCK	0.258411	0.004513
	22	PRDM2	0.253029	0.039358
	23	EZH1	0.247289	0.041796
	24	DOT1L	0.247271	0.005788
	25	KDM5A	0.231142	0.000629
	26	NEK9	0.228865	4.66E-07
	27	DTX3L	0.20794	0.020986
	28	STK4	0.198619	0.0039
	29	SETDB1	0.185543	0.037717
	30	KDM2A	0.182431	0.000208
	31	EP300	0.181673	0.005554
	32	PAK2	0.174658	0.006423
	33	HDAC1	0.162264	0.000554
	34	ASH1L	0.155677	0.043692
	35	HDAC2	0.145024	0.040182

AGS with EBV infection *in vitro*	1	STK10	0.513809	0.004313
	2	KAT2B	0.426277	0.098805
	3	SIRT2	0.375766	0.058925
	4	KDM6B	0.360451	0.009886
	5	KDM5B	0.346633	9.23E-06
	6	KDM4B	0.341718	0.015036
	7	CDK17	0.305597	0.044187
	8	NCOA3	0.302714	0.000584
	9	KDM4A	0.284234	0.015552
	10	RNF40	0.164415	0.092196
	11	KDM5A	0.160764	0.083613

Notes: Integrative analysis of single-cell transcriptome data from NPC samples and RNA-seq data from EBV-infected cell models showing the upregulated histone modifiers in NPC and GC cells upon EBV infection.

Supplementary Table 2. Clinical characteristics of NPC patients (n=120)

ID	Sex, male=1, female=2	Age	Clinical stage	Survival time (month)	Death, no=0, yes=1	Metastasis/ Recurrence, no=0, yes=1	EBV copy number	KDM5B IHC score	PLK2 IHC score
1	2	34	4a	94	0	0	540	12	12
2	1	40	3	91	0	0	373	12	12
3	1	43	4a	83	1	1	337	12	12
4	1	37	4	26	0	1	244	12	12
5	1	52	4a	95	0	0	127	12	8
6	2	50	3	94	0	0	68.7	12	8
7	1	29	4	94	0	0	54.2	12	8
8	2	30	4	92	0	0	38.4	12	8
9	1	51	3	86	0	0	37.1	12	8
10	1	34	4	84	0	0	26.7	12	8
11	1	65	3	79	0	0	20.8	12	8
12	1	49	4a	61	0	0	19.9	12	8
13	1	40	3	58	0	0	17	12	8
14	2	53	4a	29	0	1	14.3	12	8
15	1	62	3	96	0	0	13.4	12	6
16	1	63	3	89	0	0	12.4	12	6
17	1	68	3	87	0	0	11.7	12	6
18	2	24	3	83	0	0	7.95	12	6
19	1	48	4a	80	0	0	5.95	12	6
20	2	35	4a	79	0	1	5	12	6
21	1	33	4	67	1	1	4.23	12	6
22	1	38	3	63	0	0	3.3	12	6
23	1	51	3	56	0	0	2.75	12	6
24	2	53	4	55	0	1	2.19	12	6
25	1	52	4	43	1	1	2.15	12	6
26	2	52	4	38	1	1	1.75	12	6
27	1	40	4	36	0	1	1.32	12	6
28	1	40	3	92	0	1	1.05	12	4
29	2	43	4	89	0	0	0.625	12	4
30	1	40	4	86	0	0	5.73	12	4
31	1	52	4a	85	0	0	0.41	12	4
32	1	58	3	84	0	0	0.4	12	4
33	2	47	4a	82	0	0	0.382	12	4
34	1	47	3	80	0	0	0.334	12	4
35	1	32	3	80	0	0	0.292	12	4
36	2	33	3	78	0	0	0.138	12	4
37	1	33	3	78	0	0	0.114	12	4
38	1	65	4a	78	0	0	0.996	12	4
39	1	49	4a	75	0	0	0.944	12	4
40	1	54	3	73	0	0	0.497	12	4
41	1	50	3	66	1	1	0.021	12	4

42	1	63	4	65	0	0	0	12	4
43	1	47	4	61	1	1	0	12	4
44	2	42	4	59	0	1	0	12	4
45	1	44	3	42	1	1	0	12	4
46	1	41	3	40	1	1	0	12	4
47	1	52	3	26	1	1	0	12	4
48	1	57	3	86	0	0	0	12	3
49	1	45	4	36	1	1	0	12	3
50	1	55	3	9	1	1	0	12	3
51	1	44	3	88	0	0	0	12	2
52	2	43	3	86	0	0	0	12	2
53	1	56	3	79	0	0	0	12	2
54	1	50	3	77	0	0	0	12	2
55	1	44	3	46	1	1	0	12	2
56	1	51	2	81	0	0	0	12	1
57	1	51	4c	70	0	0	14	10	12
58	1	43	4	83	0	0	3.96	10	9
59	1	38	4	87	0	0	119	9	12
60	2	54	4	85	0	0	1.94	9	12
61	1	44	4a	84	0	0	0.51	9	12
62	1	37	3	92	0	0	0	9	8
63	1	44	4	86	0	0	0	9	8
64	1	45	4	75	0	0	0	9	8
65	2	36	3	73	0	0	0	9	4
66	2	56	3	63	0	0	0	9	3
67	2	33	3	59	0	0	0	9	3
68	1	63	4a	95	0	0	103	8	12
69	2	42	4	84	0	1	22.5	8	12
70	1	40	3	80	0	0	0.784	8	12
71	1	45	3	77	0	0	0.675	8	12
72	1	34	3	73	0	0	0.576	8	12
73	1	33	3	53	0	0	0	8	12
74	1	31	2	40	1	1	0	8	9
75	1	52	3	85	0	0	0	8	6
76	1	58	3	74	0	0	0	8	6
77	2	47	4	95	0	0	1.73	7	12
78	1	46	4a	97	0	0	687	6	12
79	1	25	3	96	0	0	3.33	6	12
80	1	63	3	95	0	0	2.69	6	12
81	2	63	3	94	0	0	1.12	6	12
82	2	50	3	85	0	0	0.411	6	12
83	2	51	3	81	0	0	2.29	6	12
84	2	63	3	94	0	0	0.176	6	9
85	2	49	4	93	0	0	0.118	6	9
86	1	44	3	87	0	0	76.4	6	8
87	1	25	2	79	0	0	53.3	6	8

88	1	49	3	55	0	0	0	6	8
89	2	29	2	95	0	0	0	6	6
90	1	41	2	85	0	0	0	6	6
91	1	26	3	84	0	0	0	6	6
92	1	32	3	71	0	0	0	6	6
93	2	50	3	68	0	0	0	6	4
94	1	44	4	57	0	1	0	6	4
95	1	53	3	94	0	0	112	4	12
96	2	49	4	81	0	1	5.44	4	12
97	2	33	3	80	0	0	3.6	4	12
98	2	49	3	77	0	0	83.3	4	12
99	1	64	4	85	0	0	0	4	8
100	1	60	1	78	0	0	0	4	8
101	2	60	3	76	0	0	0	4	8
102	1	52	2	55	1	1	0	4	8
103	2	62	4	67	0	1	0	4	6
104	1	56	3	75	0	0	0	4	4
105	1	68	4	85	0	0	52.7	3	12
106	2	55	3	80	0	0	13.5	3	12
107	2	30	3	79	0	0	7.77	3	12
108	1	56	4	78	0	0	3.84	3	12
109	2	45	3	63	1	1	0	3	9
110	1	45	2	78	1	1	0	3	6
111	1	54	4	86	0	0	19.6	2	12
112	1	40	3	85	0	1	3.83	2	12
113	2	33	4	83	0	0	1.92	2	9
114	2	57	2	85	0	0	0.545	2	8
115	2	43	2	74	0	0	0.147	2	6
116	1	35	3	65	0	0	0	2	6
117	1	20	4a	91	0	0	174	1	12
118	1	49	4a	81	0	0	75.9	1	12
119	2	45	4	82	0	0	17.9	1	8
120	2	41	3	55	0	1	0	1	3

Supplementary Table 3. Clinical relevance of KDM5B in NPC patients.

Groups	Low expression (n=62)	High expression (n=58)	Test of Significance
Gender			
Male	35	45	
Female	27	13	*P*=0.0141*
Age			
< 50	39	33	
≥50	23	25	*P*=0.5021
Metastasis			
0	55	42	
1	7	16	*P*=0.0355*
Stage			
1	4	1	
2	9	5	
3	32	30	
4	17	22	*P*=0.3183

Notes: Statistical analysis showing the relationship between the protein level of KDM5B and the clinicopathological characteristics of NPC patients described in Supplementary Table 2. *$P < 0.05$.

Supplementary Table 4. Antibody list.

Protein	Usage	Company	Cat NO.
KDM5B	WB/ChIP	Cell Signaling Technology	15327
KDM5B	IHC	Sigma	HPA027179
EBNA1	WB/ChIP/IP	Santa Cruz	sc-81581
BZLF1	WB/ChIP	Santa Cruz	sc-53904
BMRF1	WB	Santa Cruz	sc-58121
CEBPB	WB/IP	ABclonal	A0711
PLK2	WB/IHC	ABclonal	A7066
AKT	WB	Cell Signaling Technology	9272
p-AKT	WB	Cell Signaling Technology	9271
P110α	WB	Cell Signaling Technology	4255
mTOR	WB	Cell Signaling Technology	2983
p-mTOR	WB/IHC	Cell Signaling Technology	5536
H3K4me3	WB/ChIP/IHC	Abcam	Ab8580
GAPDH	WB	Abcam	Ab128915
Tubulin	WB	Cell Signaling Technology	2144
Actin	WB	Abclonal	AC004
Anti-mouse IgG	WB	Cell Signaling Technology	7076
Anti-rabbit IgG	WB	Cell Signaling Technology	7074

Supplementary Table 5. Sequence information for primers and small RNAs used in this study.

Target gene	sequence（5'-3'）
KDM5B-F	CCATTAGGCCGACAGTGTGT
KDM5B-R	ACATCAGCCTTGGAAGCCAT
EBNA1-F	CGTTTGGGAGAGCTGATTCT
EBNA1-R	CCCCTCGTCAGACATGATTC
LMP1-F	CCCTTTGTATACTCCTACTGATGATCAC
LMP1-R	ACCCGAAGATGAACAGCACAAT
LMP2A-F	CAATGGCGACCGTCACTC
LMP2A-R	TCCTCTGCCCGCTTCTTC
BZLF1-F	CATGTTTCAACCGCTCCGACTGG
BZLF1-R	GCGCAGCCTGTCATTTTCAGATG
BRLF1-F	GAAGCCCGGTGCCCAAAG
BRLF1-R	GTGTCACTGTTGCCCGAGTC
BMRF1-F	ACCTGCCGTTGGATCTTAGTG
BMRF1-R	GGCGTTGTTGGAGTCCTGTG
BcLF1-F	GTGGATCAGGCCGTTATTGA
BcLF1-R	CCTCAAACCCGTGGATCATA
CEBPB-F	GCCCTCGCAGGTCAAGAGCA
CEBPB-R	TTGAACAAGTTCCGCAGGGTG
SOX2-F	TACAGCATGTCCTACTCGCAG
SOX2-R	GAGGAAGAGGTAACCACAGGG
NANOG-F	CCCCAGCCTTTACTCTTCCTA
NANOG-R	CCAGGTTGAATTGAACCAGGTC
OCT4-F	CTGGGTTGATCCTCGGACCT
OCT4-R	CCATCGGAGTTGCTCTCCA
ABCG2-F	CAGGTGGAGGCAAATCTTCGT
ABCG2-R	ACCCTGTTAATCCGTTCGTTTT
BMI-1-F	GCTGCCAATGGCTCTAATGAA
BMI-1-R	TGCTGGGCATCGTAAGTATCTT

FAT4-F	TAGCACCAGACAGGGCTACT
FAT4-R	CCACCAGGTTGATCACGTCG
FHL2-F	CGCTTTGACTGCCACCATTG
FHL2-R	TGCATTCCTGGCACTTGGAT
GCLM-F	GGAACCTGCTGAACTGGGG
GCLM-R	CCATGTCAACTGCACTTCTAGT
JPH2-F	GATGATGGAGGGGCGTACTG
JPH2-R	CCAGGTGCCCTCATACTTGG
IL17RE-F	CTCCTGCCTCTCCTCCTCAT
IL17RE-R	GCTGGCATCTTGTGTCTCCT
SH3RF2-F	TGCCCTGTGTGCTTTGAGAA
SH3RF2-R	CACTAGCCGGAAAGGACTGG
PLK2-F	GCGGACTATCACCTACCAGC
PLK2-R	AGACACAATCTGCCTGAGGT
TPM4-F	AAGGAGAATGCCATCGACCG
TPM4-R	TTTCTGCCTCCTCCAGCTTC
FAT4-ChIP-F	TTCCATCGCCTCCAGCTTTT
FAT4-ChIP-R	GCCAAAACACTCGAAGGAGC
FHL2-ChIP-F	ACTGTGGCTGAGAACTGTGT
FHL2-ChIP-R	ACACTCCTCGCAGGTGTTG
GCLM-ChIP-F	TTGGAATCAGCTCTCCCGC
GCLM-ChIP-R	ACAATCATGAAGCTCCTCGC
JPH2-ChIP-F	ACTGGAGGAGTGTTGCAGG
JPH2-ChIP-R	AGACACCTGCCACCTCAAAG
IL17RE-ChIP-F	AAGCCCAGCAGCTGATTCTT
IL17RE-ChIP-R	AAGAGGTGCTTCCA GGCAAA
SH3RF2-ChIP-F	AGCCCTGGTCTCTACACCA
SH3RF2-ChIP-R	GGTCTCTGCAGGGATCCATT
BS#1-F	CTTCAGGGCAGGAACTCTGA
BS#1-R	TCAGAGTTCCTGCCCTGAAG
BS#2-F	CAAGTCTTTCGCCCCTCTCA

BS#2-R	AGCATGTTCATGAGAAGGCA
BS#3-F	CCCCTTTTAATTGAAGACTTCT
BS#3-R	CCTCATTTTGCAGAGCAGGC
PLK2-NheI-F	CTAGCTAGCATGGAGCTTTTGCGGACTATC
PLK2-BamHI-R	CGCGGATTCTCAGTTACATCTTTGTAAGAGC
KDM5B-Luc-XhoI-F	CCGCTCGAGCCCGTTGTGTATTTCTACCTGT
KDM5B-Luc-HindIII-R	CCCAAGCTTAACAGCAAGTCCGAGTTGTAC
PLK2-Luc-KpnI-F	CGGGGTACCAACCAGGGCAGCTCATGAGCC
PLK2-Luc-NheI-R	CTAGCTAGCGAGTGAGAGCGCTCGGCGA
CEBPBmt#1-F	ATTAGAACAGTGCCTCTTCGCGACCGATGCCCCAAAATGTTA
CEBPBmt#1-R	TAACATTTTGGGGCATCGGTCGCGAAGAGGCACTGTTCTAAT
CEBPBmt#2-F	TGTGAATTATGGCACCGGATCCGGACTACCTATGCCTCAC
CEBPBmt#2-R	GTGAGGCATAGGTAGTCCGGATCCGGTGCCATAATTCACA
JUNDmt#3-F	AAATTGCTTAACCTATTCGATTGCAGACCCTCTTATTGTAAAGTGG
JUNDmt#3-R	CCACTTTACAATAAGAGGGTCTGCAATCGAATAGGTTAAGCAATTT
ZRE1mt-F	AGTCTCCATCACTTACCGATTCTGTGAATTATGGCAA
ZRE1mt-R	TTGCCATAATTCACAGAATCGGTAAGTGATGGAGACT
ZRE2mt-F	AGTATCTTCTTCCCTCATAGCCCTCGCCTTCTGTATA
ZRE2mt-R	TATACAGAAGGCGAGGGCTATGAGGGAAGAAGATACT
KDM5B-siRNA#1	GCGTATCCGTTTGGAACAA
KDM5B-siRNA#2	CTACGGCAGTAAAGGAAAT

PLK2-siRNA#1	GTAGAAGGTCAATGGCTCATA
PLK2-siRNA#2	GTGACGGTGCTGAAATACTTT
CEBPB-siRNA	CCCGTGGTGTTATTTAAAGAA
sh luci-F	CCGGTTCCTGGAACAATTGCTTTTACTCGAGT AAAAGCAATTGTTCCAGGAATTTTTG
sh luci-R	AATTCAAAAATTCCTGGAACAATTGCTTTTA CTCGAGTAAAAGCAATTGTTCCAGGAA
KDM5B-shRNA-1-F	CCGGAAGCGTATCCGTTTGGAACAACTCGAG TTGTTCCAAACGGATACGCTTTTTTTG
KDM5B-shRNA-1-R	AATTCAAAAAAAGCGTATCCGTTTGGAACAA CTCGAGTTGTTCCAAACGGATACGCTT
KDM5B-shRNA-2-F	CCGGAACTACGGCAGTAAAGGAAATCTCGA GATTTCCTTTACTGCCGTAGTTTTTTTG
KDM5B-shRNA-2-R	AATTCAAAAAAACTACGGCAGTAAAGGAAA TCTCGAGATTTCCTTTACTGCCGTAGTT
EBNA1-DBD-DELE-F	AAGGCCCAAGCACTGGACCCCGGTTTGGAAT GGCCCCTGGACC
EBNA1-DBD-DELE-R	GGTCCAGGGGCCATTCCAAACCGGGGTCCAG TGCTTGGGCCTT
